# Construction of Peptide Amphiphile-Coated Coacervates with Selective Permeability

**DOI:** 10.1021/acsbiomaterials.5c02101

**Published:** 2026-02-17

**Authors:** Bin Wang, Kristi L. Kiick, Millicent O. Sullivan

**Affiliations:** † Department of Materials Science and Engineering, 5972 University of Delaware, Newark 19716, Delaware, United States; ‡ Department of Biomedical Engineering, University of Delaware, Newark 19716, Delaware, United States; § Department of Chemical and Biomolecular Engineering, University of Delaware, Newark 19716, Delaware, United States

**Keywords:** peptide amphiphile, coacervate, self-assembly, charge interactions, coating, surface modification, formulation, selective permeability

## Abstract

The combination of membranes with coacervates has been regarded as an effective approach to stabilize coacervates and modify their surface properties. Here, we achieved the construction of a functional coacervate system by localizing nanovesicles assembled by elastin-like peptide-block-collagen-like peptides (ELP-CLPs) on the surface of polyelectrolyte coacervates. The formation of the ELP-CLP coating was driven by electrostatic interactions between negatively charged ELP-CLP vesicles and positively charged coacervates. Altering the surface charge of ELP-CLP vesicles or coacervates disrupted the formation of coatings, and the formulation parameters, such as different mixing protocols and the order of adding the components, could be used to control the coating process. The ELP-CLP vesicle coating successfully functionalized the coacervates and presented the ability to control the diffusion of molecules based on their different molecular weights. Our results demonstrated approaches to control the coating process and coating functionality of ELP-CLP vesicle coatings and highlighted their potential application as a novel surface modification to provide selective permeability to current coacervate systems.

## Graphical abstract

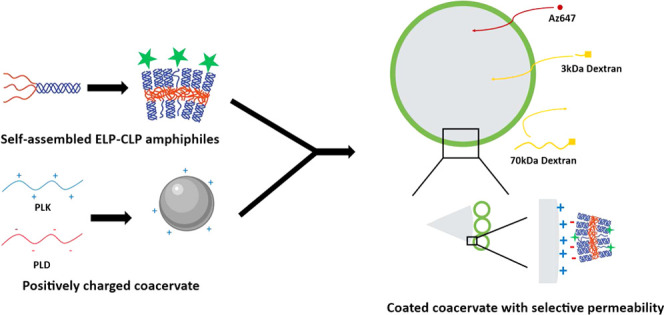

## Introduction

Developing materials that mimic the structure of natural organelles has garnered attention as an attractive approach to study cell behavior,^1,2^ model or house bioreactions in vitro,^3,4^ create structures able to incorporate catalytically active compartments for biotech applications,^5^ and introduce novel functionalities into live cells.^6,7^ The self-assembly of lipid vesicles has been widely applied to create compartments that encapsulate active biomolecules within a membrane. These liposome-based systems offer advantages due to their ability to provide moderate aqueous conditions for delicate biomolecules such as enzymes and RNAs.^8,9^ However, liposome-based structures offer limited permeability for large molecules, present difficulties in encapsulating functional molecules, such as enzymes, and exhibit limitations in their capacity to mimic the viscous, crowded environment of the native cytosol. To overcome such disadvantages, the formation of droplets (e.g., hydrogels from emulsion templating or microfluidic templating^10^ and coacervates from liquid–liquid phase separation (LLPS)^11,12^ ) has been applied as an alternative approach to construct compartments with a high-viscosity, electrolyte-dense chemical environment. These droplet structures are promising candidates for stabilizing concentrated biomolecules and housing biochemical reactions in vitro.^13–15^ However, simple coacervate systems lack physical boundaries on their surface and, accordingly, offer limited control over the exchange of molecules between the coacervate droplet and the surrounding environment.^16,17^

Recent studies have successfully combined membranes or membrane vesicles with coacervate droplets to create hybrid structures with unique properties. This combination has been achieved through various strategies, such as localized LLPS in lipid giant unilamellar vesicles (GUVs),^18,19^ templated lipid bilayer assembly on the surface of coacervates,^20,21^ selective LLPS with nucleation sites on lipid membranes,^22,23^ and penetration of coacervates into lipid vesicles.^24,25^ Membrane-coated coacervates exhibit ideal properties for a variety of biotechnological and synthetic cell applications by virtue of their capacity to mimic the condensed environment and controlled permeability of membrane organelles. Furthermore, such functional coacervates offer fundamentally important properties for understanding membrane–coacervate interactions in cells and designing drug delivery carriers. However, the low stability of lipid bilayers hinders the reliability of the formulation process for producing lipid membrane-coated coacervates.^26,27^ Furthermore, although the rigid bilayer structure of liposomes has been shown to perform well in compartmentalizing different coacervates, the limited diffusion through lipid bilayers has restricted the transfer of substrates and products between the surrounding environment and the functional coacervate core.^25^ While only small nonpolar molecules (e.g., O_2_ and CO_2_) are highly permeable in lipid bilayers,^28^ most coated coacervates composed of lipids require the pre-encapsulation of necessary components, and the final product is not readily released. While natural membranes facilitate the insertion of membrane proteins to control molecular transfer, recreating the complex functionality of natural membranes through artificial insertion and stabilization of membrane proteins in artificial lipid bilayers remains challenging.^29,30^

To provide control of membrane permeability and facilitate the design of membrane structures with diverse properties, amphiphilic polymers have been applied as alternative building blocks to construct coated coacervates.^31^ Several studies have applied amphiphilic polymers to encapsulate various catalysts (e.g., metal capsules,^32,33^ inorganic particles,^34^ and organic polymers^35,36^ ) and achieved compartmentalization and stabilization of the encapsulated cargoes from the environment.^37,38^ Such functional coacervates, however, require specific polymer sequences and chain lengths and have exhibited an inability to control molecular diffusion across the boundary. Therefore, there is a clear demand for the development of novel systems with controlled permeability that combine the advantages of lipid bilayers, such as adaptability to various coacervate systems, with stable assembly and robust formulation, offered by amphiphilic polymers.

In our recent studies, amphiphilic peptide-based materials comprising elastin-like peptides (ELPs) have shown sequence-dependent and tunable thermal responsiveness as well as controlled self-assembly properties.^39,40^ The formation of ELP-based nanovesicles comprising a bilayer structure (ca. 20 nm thickness) highlighted amphiphilic polypeptides as a novel membrane system that applied stable polymeric amphiphiles to mimic the structure of phospholipids and their self-assembled bilayers. Modification of the hydrophilic peptide domain (e.g., collagen-like peptides, or CLPs, or bundle-forming peptides, BFPs) offered a pathway to change the interlayer interactions and thereby tune the higher-ordered assembly of ELP-based amphiphiles from simple vesicles to multilayer vesicles.^41,42^ Furthermore, decreasing the hydrophobic ELP length or increasing the hydrophilic CLP length induced a morphological transition in the ELP-CLP amphiphile assemblies from vesicles to plate-like structures, demonstrating the versatility of the ELP-CLP platform. These vesicles also have shown the ability to encapsulate both hydrophilic cargoes (e.g., vancomycin) and hydrophobic cargoes (e.g., fluorescein), and the loaded cargoes can be released simultaneously.^43,44^

Due to the stable assembly of ELP-CLP conjugates into vesicles under physiologically relevant solution conditions, we hypothesized that ELP-CLPs could serve as a novel building block to apply amphiphilic bilayers in the construction of all-protein artificial membrane organelles. Herein, we demonstrate the construction of functional coacervates by coating polyelectrolyte coacervates composed of poly-l-lysine (PLK-100) and poly-l-aspartic acid (PLD-100) with the self-assembled ELP-CLP vesicles. Dynamic light scattering (DLS) was used to confirm the successful self-assembly of the ELP-CLP vesicles, and zeta potential (ZP) measurements were used to analyze the surface charges of the coacervate droplets and ELP-CLP vesicles prior to the coating process. Following the coacervate coating, confocal microscopy was used to visualize the location of fluorescent-dye-labeled ELP-CLP molecules in the hybrid structure. We investigated key parameters controlling the coating process, including the effect of changing the relative concentrations of ELP-CLP vs. coacervate, the surface charges of the coacervate droplets and ELP-CLP vesicles, and the order of addition of vesicle to coacervate or vice versa. In addition, diffusion experiments using fluorescent model molecules of various molecular weights were performed via confocal fluorescence microscopy to characterize the selective permeability of the ELP-CLP coating. Our results demonstrated a novel charge-driven pathway to create a functional coating on the surface of coacervates with amphiphilic polypeptides.

## Results and Discussion

The scheme for the formulation of ELP-CLP-coated coacervates is shown in Figure 1. ELP-CLPs were synthesized by conjugating C-terminal alkyne-functionalized ELPs [(VPGFG)_6_, abbreviated as F_6_] and N-terminal azide-functionalized CLPs with a functional residue/group at the C-terminus [including the unmodified CLP (GPO)_7_GG (O for hydroxyproline) with an amide C-terminus, abbreviated as G_7_GG; the negatively charged CLP (GPO)_7_GG carrying a carboxyl group at the C-terminus, abbreviated as G_7_GG-COOH; and the neutral CLP-cysteine (GPO)_7_GC with an amide C-terminus, abbreviated as G_7_GC]. All peptides were synthesized via solid phase peptide synthesis (SPPS) (Figures S1–S3). Conjugated products included unmodified ELP-CLP (F_6_-G_7_GG), negatively charged ELP-CLP (F_6_-G_7_GG-COOH), and Cys-labeled ELP-CLP (F_6_-G_7_GC) (Figures S4–S6). Functionalized ELP-CLP vesicles (charged or Cys labeled) were prepared by mixing 2 mg/mL of unmodified ELP-CLP (F_6_-G_7_GG) and 2 mg/mL of modified ELP-CLP (F_6_-G_7_GG-COOH for charged ELP-CLP vesicles or F_6_-G_7_GC for Cys-labeled ELP-CLP vesicles) at a 9:1 volume ratio. These subsets of ELP-CLPs were then labeled for visualization via confocal microscopy with a fluorescent dye (Az488), using the thiol–maleimide reaction (for Cys-labeled ELP-CLP vesicles) or the EDC/NHS reaction (for charged ELP-CLP vesicles). The ELP-CLP vesicle solutions were further concentrated 3 times to reduce the effect of dilution on the coacervates after adding ELP-CLP vesicle solution. The characterization of ELP-CLP vesicles after the labeling reaction is shown in Figures S7 and S8. These results confirmed the successful labeling of ELP-CLP, and no significant change of ELP-CLP vesicles’ surface charge or particle size after labeling was observed.

**Figure 1:**
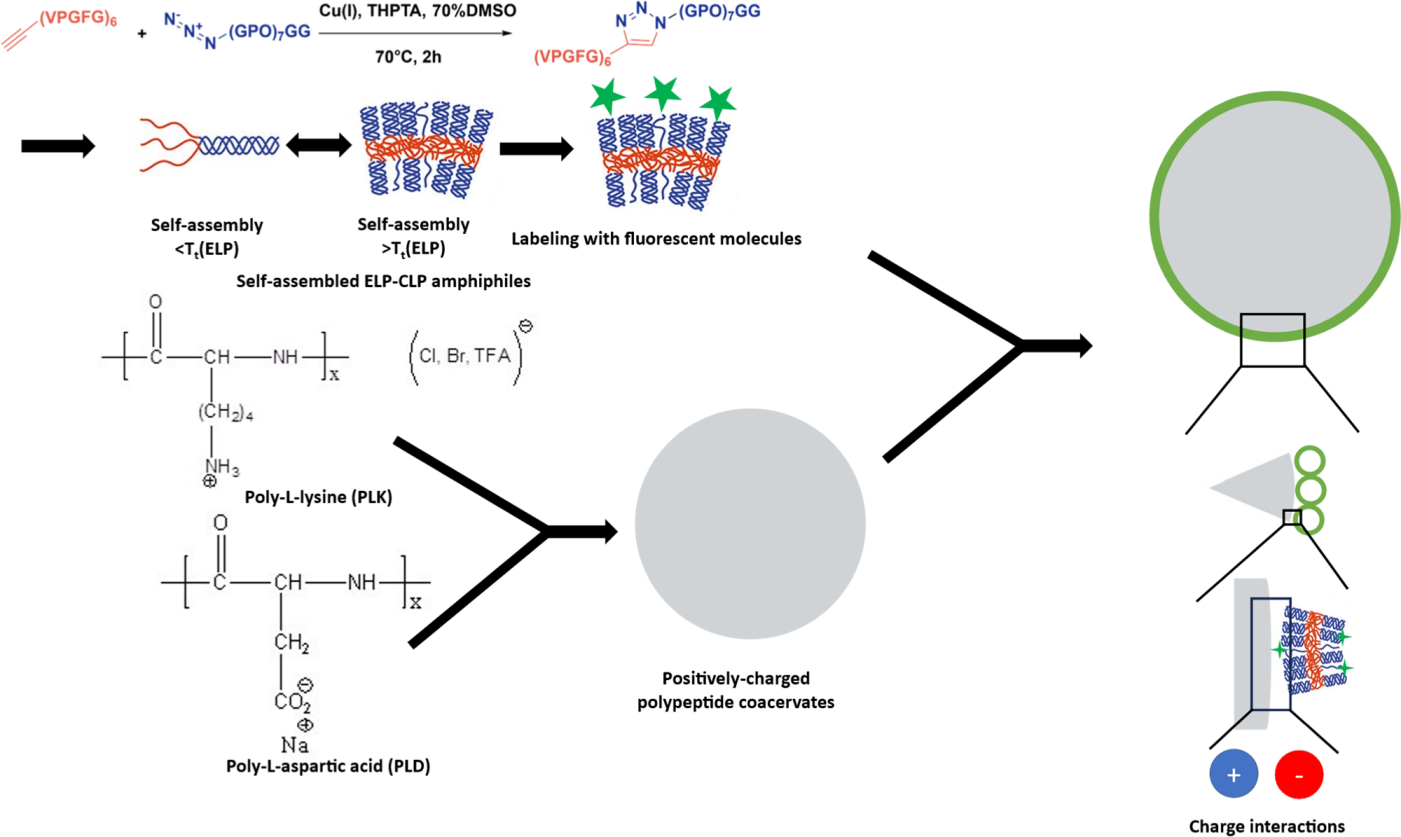
Scheme of the construction of the ELP-CLP-coated coacervates.

The charged PLK/PLD coacervates were freshly prepared in a 50 mM pH 6.8 MES buffer. The surface charge of the coacervate varied by different batches and ratios of PLK and PLD stock solutions and was determined by measuring the zeta potential. The zeta potential of coacervates was confirmed to be above 30 mV (for positively charged coacervate, abbreviated as p-coacervate) or less than −30 mV (for negatively charged coacervate, abbreviated as n-coacervate) for different experiments (Table S1). The charged coacervate was then gently mixed with the labeled ELP-CLP vesicle solution at different ratios by manual agitation and left on the benchtop for at least 30 min before imaging.

The influence of the ELP-CLP vesicle concentration on the surface coating of the positively charged coacervate (p-coacervate) was first investigated. Different ratios of the negatively charged, labeled ELP-CLP vesicles were added to the solution with preformed positively charged coacervates. Without ELP-CLP vesicles, coacervates were formed as expected due to the electrostatic interactions between PLD and PLK (Figure 2A). When a 2 vol % [12 mol %, maximum ELP-CLP: (PLK + PLD) molar ratio calculated by the initial ELP-CLP concentration; lower ratio is expected in the mixture solution due to the disassembly of ELP-CLP vesicles during the labeling process] ELP-CLP vesicle solution was added to the p-coacervate solution, only fluorescent aggregates were observed, and no increased fluorescence intensity was detected on the surface of the coacervate (Figure 2B). These results suggested that ELP-CLP vesicles at low concentrations did not form any coating on the surface of the coacervates and that the fluorescent aggregates may be attributed to the formation of ELP-CLP aggregates during the labeling process. When the ELP-CLP vesicle concentration was increased to 5 vol % (30 mol %), increased fluorescence intensity was observed on the surface of the coacervates. However, these fluorescent shells were heterogeneous and only a fraction of the coacervates were found to be coated (Figure 2C). In this instance, imaging indicated that the ELP-CLP vesicles started localizing on the surface of the coacervate droplets, forming incomplete coatings. When the ELP-CLP vesicle solution concentration was further increased to 10 vol % (60 mol %), a homogeneous coating was found on the surface of coacervates (Figure 2D). The formation of the ELP-CLP coating with increased ELP-CLP concentration suggested the existence of a minimum concentration for ELP-CLP to coat the coacervate. Interestingly, the increase in the apparent coacervate size and the slightly increased fluorescence intensity inside the particle (Figures S9A and 2E) suggested that the addition of the ELP-CLP vesicles promoted some aggregation of the p-coacervates. This concentration-dependent behavior further suggested that the interactions between the coacervates and ELP-CLP vesicles were different at different ELP-CLP vesicle concentrations (Figure 2F). With a low concentration of ELP-CLP vesicles (2 vol %), the ELP-CLP promoted the aggregation of coacervate, thus producing a larger coacervate. With higher ELP-CLP vesicle concentration (5 vol % and 10 vol %), the aggregated coacervates presented higher surface charge density, which prevented further aggregation of these coacervates, and due to the charge–charge interactions between the positively charged coacervates and the negatively charged ELP-CLP vesicles, the ELP-CLP bound on the surface of the coacervates resulted in the gradual formation of the ELP-CLP coating. Cryogenic scanning electron microscopy (cryo-SEM) studies were also conducted to visualize the differences between the surfaces of the coacervate droplets in the absence or presence of ELP-CLP vesicles (Figure S9B,C). In samples lacking ELP-CLP, amorphous ice was observed on the surface of the coacervates, and no significant difference was observed between the interior and exterior of the coacervates (from the cross section of the coacervate). However, for the ELP-CLP-coated coacervates, small particles were observed on the surface of the coacervates with rough diameters that suggest a coating with the ELP-CLP vesicles. In addition, the image of the cross section of the ELP-CLP-coated coacervates showed different contrasts on the surface of the coacervate versus the interior of the coacervate, suggesting the formation of a coating on the surface that is different from the surface of the p-coacervates alone.^45,46^

**Figure 2:**
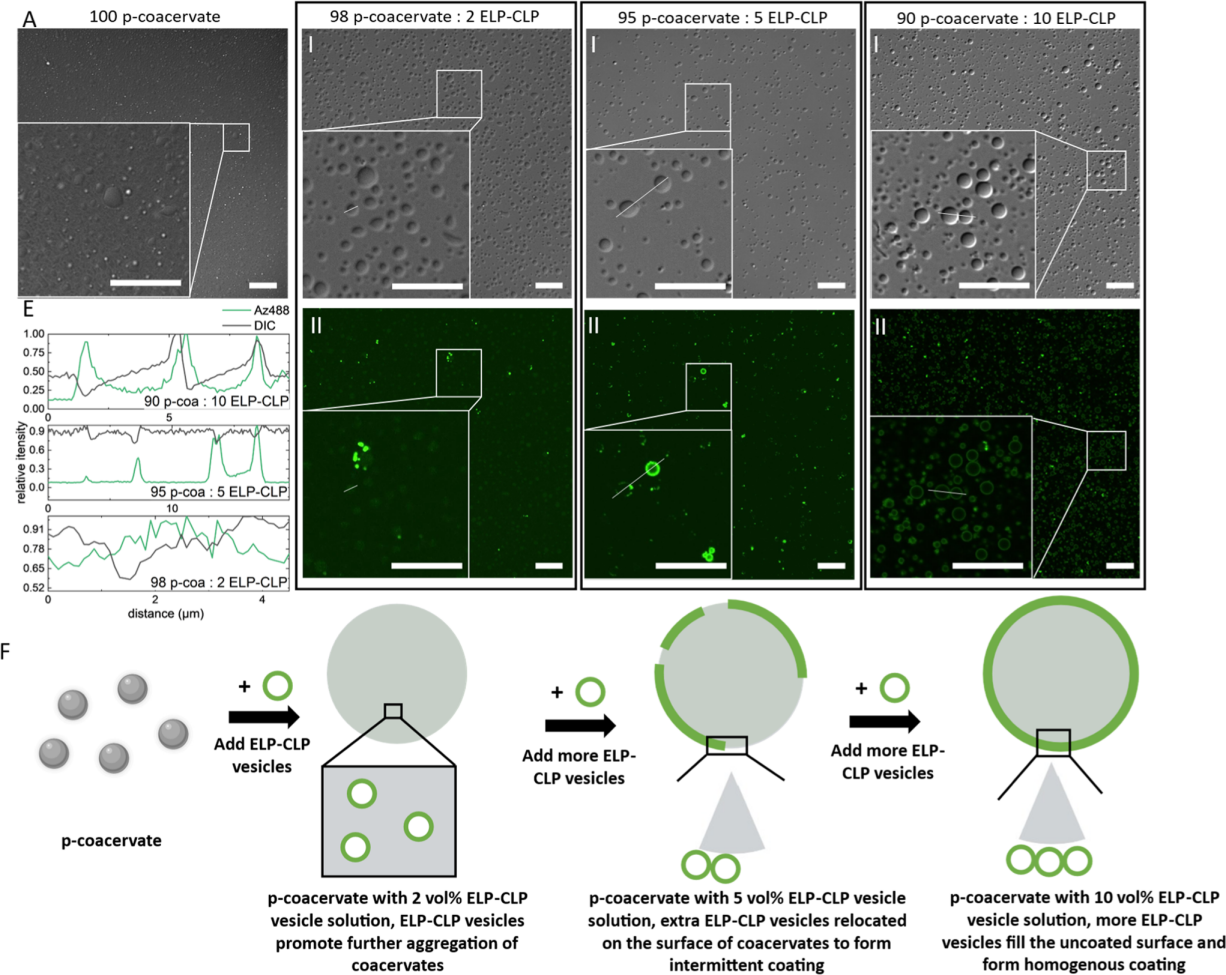
(A) Differential interference contrast (DIC) channel images of p-coacervate. (B) (I) DIC and (II) fluorescence channel images of p-coacervate after coating with 2 vol % labeled ELP-CLP solution. (C) (I) DIC and (II) fluorescence channel images of p-coacervate after coating with a 5 vol % labeled ELP-CLP solution. (D) (I) DIC and (II) fluorescence channel images of p-coacervate after coating with a 10 vol % labeled ELP-CLP solution. (E) The intensity map of highlighted white lines in confocal images indicates that no membrane formed in the 2 vol % ELP-CLP sample, an intermittent membrane formed in the 5 vol % ELP-CLP sample, and a membrane formed in the 10 vol % ELP-CLP sample. The relative intensity is defined as the ratio between the local intensity and maximum intensity in the selected area. (F) Proposed scheme of dynamic interactions between ELP-CLP vesicles and p-coacervates. ELP-CLP vesicles first promote aggregation of coacervates and then gradually form a coating on the coacervates. All scale bars = 20 μm.

One important feature of lipid-coated artificial organelles is their ability to compartmentalize and stabilize encapsulated materials and prevent them from interacting with the solution. To probe the properties of coacervates coated with ELP-CLP vesicles, an aging experiment with ELP-CLP-vesicle-coated coacervates was conducted. In the absence of ELP-CLP vesicles, the coacervate was redissolved in solution in less than 1 day based on the observation of decreased turbidity and the disappearance of the coacervates under confocal microscopy (Figure S10). With 2 vol % ELP-CLP, we observed that the coacervate solution remained turbid after 1 day, unlike the uncoated samples. The 2 vol % solutions turned into a clear solution without any precipitation after 1 week. Furthermore, the coated coacervate was not redissolved after 1 week based on our ability to detect coacervates through confocal imaging (Figure 3A). In addition, imaging showed that the ELP-CLP was still localized on the surface of the coacervates and did not diffuse into the coacervate, indicating the formation of stable coated coacervates and the ability of ELP-CLP to compartmentalize and stabilize PLK/PLD coacervates in solution.

**Figure 3:**
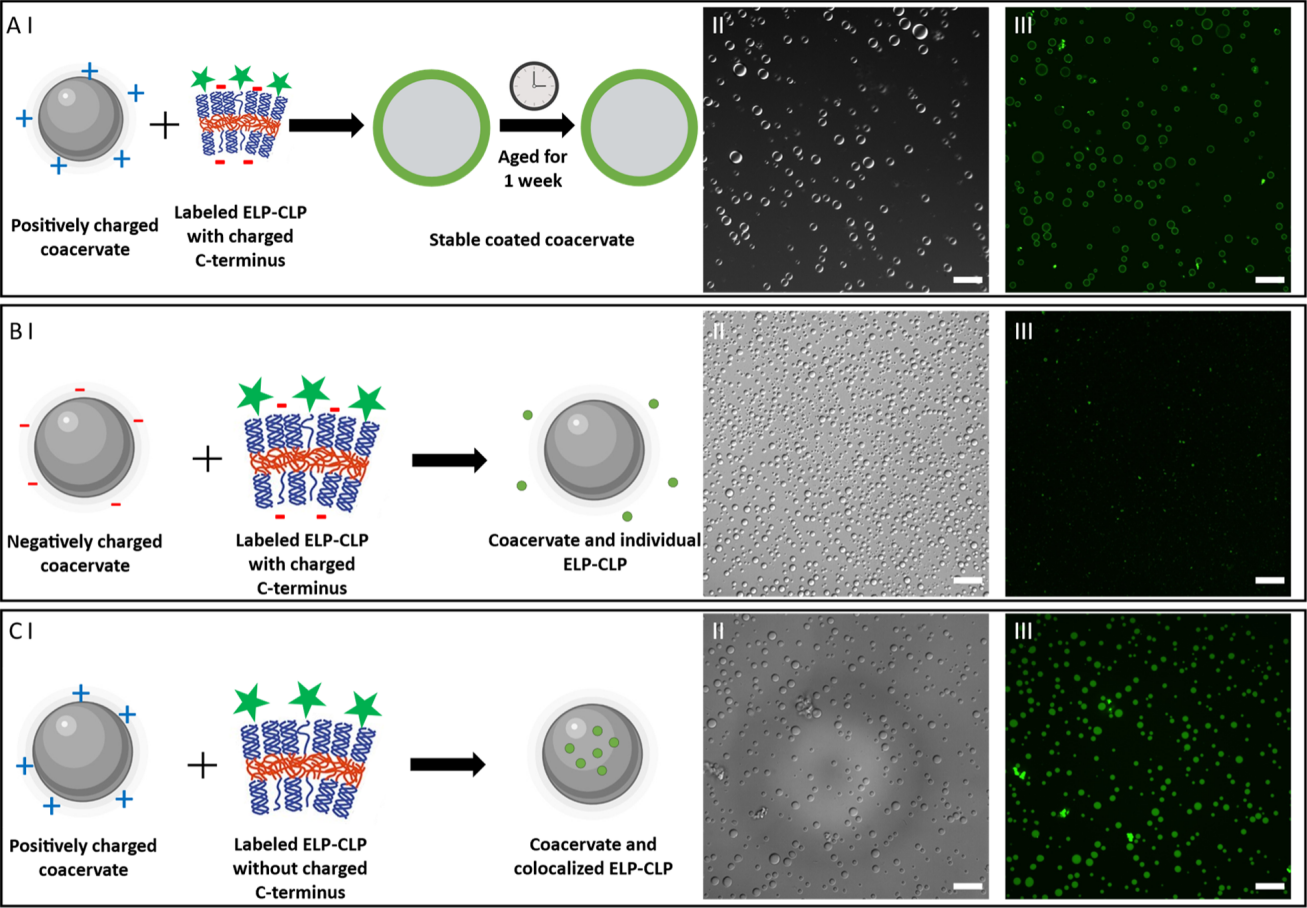
(A) (I) Coated coacervates formed by mixing p-coacervate and charged ELP-CLP with 1 week aging and confocal images in (II) DIC and (III) fluorescence channels. (B) (I) Isolated coacervates and ELP-CLP aggregates formed by mixing n-coacervate and charged ELP-CLP and confocal images in (II) DIC and (III) fluorescence channels. (C) (I) Coacervates and colocalized ELP-CLP aggregates formed by mixing p-coacervate and uncharged ELP-CLP and confocal images in (II) DIC and (III) fluorescence channels. All scale bars = 20 μm.

Since charge–charge interactions are expected to play a crucial role in facilitating the localization of ELP-CLP vesicles to the surface of the coacervates, the influence of coacervates and ELP-CLP surface charge was also investigated. We first changed the surface charge of coacervates to negative by increasing the amount of PLD in the coacervate while maintaining the ELP-CLP vesicle charge. When negatively charged coacervates were mixed with negatively charged ELP-CLP vesicles, there was no fluorescence signal colocalized with the coacervate and fluorescent aggregates were found to be randomly distributed (Figure 3B). This result indicated that ELP-CLP was distributed in aqueous solution and showed no preference for interacting with the coacervates due to the electrostatic repulsion between the coacervates and the ELP-CLP vesicles, which prevented the coating process and prohibited the diffusion of ELP-CLP vesicles into the coacervates. Another set of experiments utilizing the positively charged coacervate and neutral ELP-CLP vesicles showed a homogeneous colocalization of fluorescence within the entire interior of the coacervate particle (Figure 3C), indicating that the ELP-CLP was concentrated inside the coacervates. This result suggested an affinity (nonelectrostatic) between the coacervate and the uncharged ELP-CLP; such interactions between polyelectrolytes and proteins have been previously reported.^47,48^ Without charge interactions between coacervates and ELP-CLP vesicles, the uncharged ELP-CLP vesicles can easily diffuse into the neutrally charged coacervate core, and the polar interactions between ELP-CLP and polyelectrolyte could promote the localization of ELP-CLP vesicles in the coacervate. However, with charged ELP-CLP vesicles, the charge interaction can either trap the ELP-CLP on the surface of the coacervate (if coacervates and ELP-CLP vesicles carry counter charges) or prevent ELP-CLP vesicles from diffusing into the coacervate due to the charge repulsion (if coacervates and ELP-CLP vesicles carry the opposite charge). The disappearance of a coacervate coating upon alteration of the charges of coacervates or ELP-CLP vesicles highlights the importance of charge interactions in the ELP-CLP coating process.

The effect of the formulation protocol on the preparation of the coacervates was also investigated. Since the formation of coacervates is a highly dynamic process and ELP-CLP was physically coated on the surface of coacervates, we hypothesized that the mixing process could be used to control the behavior of the ELP-CLP vesicle interactions with the coacervate. We applied a more intense mixing procedure by vortexing (instead of manual agitation) the preformed coacervate and ELP-CLP vesicles for 30 s. An increased fluorescence signal could still be observed on the surface of the coacervates as compared to the inside or outside of the coacervates (Figure 4A), suggesting that the coating of ELP-CLP vesicles on the p-coacervate remained intact even after an intense mixing process. To compare the different ELP-CLP behaviors among different formulation protocols, relative intensity was used to compare the signals inside vs. on the surface of the coacervates. Defined as the ratio between the local fluorescence intensity and highest fluorescence intensity in the selected area, the relative intensity reflected the different ratios of ELP-CLP vesicles that participated in the coacervate aggregation process (localized inside the coacervate) or the coating process (localized on the surface of the coacervates). Compared to coated coacervates prepared by gentle mixing (Figure 2D,E), higher fluorescence intensity (about 0.5 relative intensity for the vortexed sample and 0.25 relative intensity for the manually agitated samples) was observed inside the coacervates (Figure 4B), indicating that more ELP-CLP was trapped in the coacervates. When the coated coacervate was prepared by mixing PLK and a premixed negatively charged ELP-CLP and PLD solution, no significant difference in the localization and intensity of fluorescence was observed in comparison to coated coacervates prepared with preformed coacervate and preformed ELP-CLP vesicles (Figure 4B,C). Since both the ELP-CLP vesicle and PLD carried negative charges, the repulsion prevented ELP-CLP vesicles and PLD from binding to each other in the premixed solution and no aggregation was observed. When PLK was added to the system, PLK and PLD interacted with each other first and formed the coacervate in the absence of ELP-CLP vesicles, and these coacervates subsequently reacted with ELP-CLP vesicles to form a coated coacervate. Thus, the premixing of the negatively charged ELP-CLP vesicles and PLD did not significantly change the coating behavior. When the ELP-CLP vesicles were premixed with PLK, an increase in fluorescence intensity was observed inside the coacervates (about 0.75 relative intensity; Figure 4B,D). The counter charge promoted the interaction of the positively charged PLK with the negatively charged ELP-CLP vesicles, which then facilitated colocalization of the partially neutralized ELP-CLP vesicles inside the coacervates formed upon the addition of the positively charged PLK. Variations in the “severity” of the formulation process and alteration in order of addition showed significant impact on the formation of ELP-CLP-coated coacervates. The ELP-CLP vesicles could be trapped in the coacervate by the application of this more intense mixing procedure or by reducing the surface charge of ELP-CLP vesicles through changes to the order of addition. These results are also consistent with our hypothesis that the ELP-CLP vesicles first promote coacervate aggregation, and then only at higher ELP-CLP concentrations form a coating on the coacervate surface (Figure 2F).

**Figure 4:**
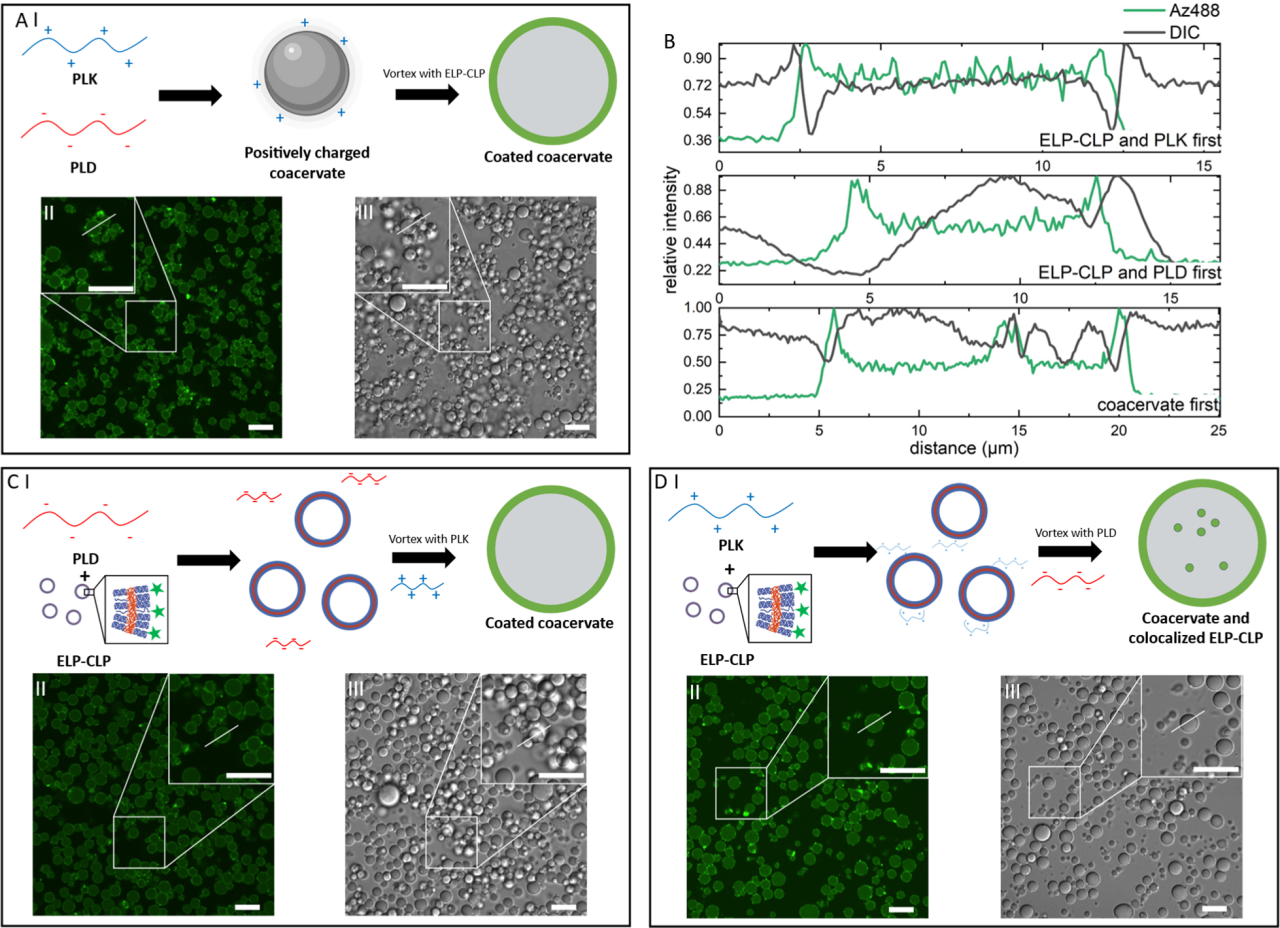
(A) (I) Coated coacervates formed by vortexing charged ELP-CLP and p-coacervate and confocal images in (II) DIC and (III) fluorescence channels. (B) The intensity map of highlighted white lines in confocal images. (C) (I) Coated coacervates formed by vortexing PLK and premixed charged ELP-CLP and PLD solution, and confocal images in (II) DIC and (III) fluorescence channels. (D) (I) Coacervates with colocalized ELP-CLP formed by vortexing PLD and premixed charged ELP-CLP and PLK solution, and confocal images in (II) DIC and (III) fluorescence channels. All scale bars = 20 μm.

Since selective permeability is crucial for applying membrane-coated coacervates in biotechnological or synthetic cell applications, experiments were carried out to test the permeability of the ELP-CLP coating. Three different hydrophilic molecules were selected to represent different types and molecular weights of molecular cargos, and these molecular cargos were added to solution with the coated coacervates to test whether they could diffuse through the ELP-CLP coating. The model molecules included a small hydrophilic dye, Az647, a low molecular weight (MW)-labeled hydrophilic polymer [Texas Red-labeled dextran (MW = 3 kDa)], and a high MW-labeled hydrophilic polymer [Texas Red-labeled dextran (MW = 70 kDa)]. The model molecules were dissolved in water and added to coacervates with or without an ELP-CLP coating. The mixtures were aged for 1 h to allow the penetration of the model molecules into the coacervates, and then the coacervates were imaged using confocal microscopy. The fluorescent signal inside or outside the coacervate was compared to determine whether there was any selective permeability of the ELP-CLP coatings. For coacervates lacking the ELP-CLP coating, increased fluorescence intensity was observed inside the coacervate for all three model molecules, indicating the colocalization of model molecules inside the coacervate (Figures 5A and S11, and Table S2). These findings were consistent with previous research that showed strong affinities between coacervates and hydrophilic molecules.^49–51^ For coacervates with the ELP-CLP coating, an increase in the fluorescence intensity of Az647 and 3 kDa dextran was observed inside the coacervates, while the fluorescence intensity for the 70 kDa dextran was greater outside of the coated coacervate (Figure 5, Table S3). In addition, both 3 and 70 kDa dextran showed a strong preference for localizing on the surface of coacervate, shown as increased fluorescence signals of labeled dextrans colocalized with the ELP-CLP coating (Figure 5D,E, Table S3). These increased dextran concentrations suggest an affinity between exposed CLP domains on ELP-CLP vesicles and dextran.^52–54^ These data indicate that the ELP-CLP coating showed molecular weight-dependent diffusion into the coacervates, since the Az647 and 3 kDa dextran could diffuse through the ELP-CLP coating, thus increasing the fluorescence signals observed inside the coacervates, while the ELP-CLP coating hindered the diffusion of 70 kDa dextran into the coacervate, consistent with a reduction in diffusion across the membrane.

**Figure 5:**
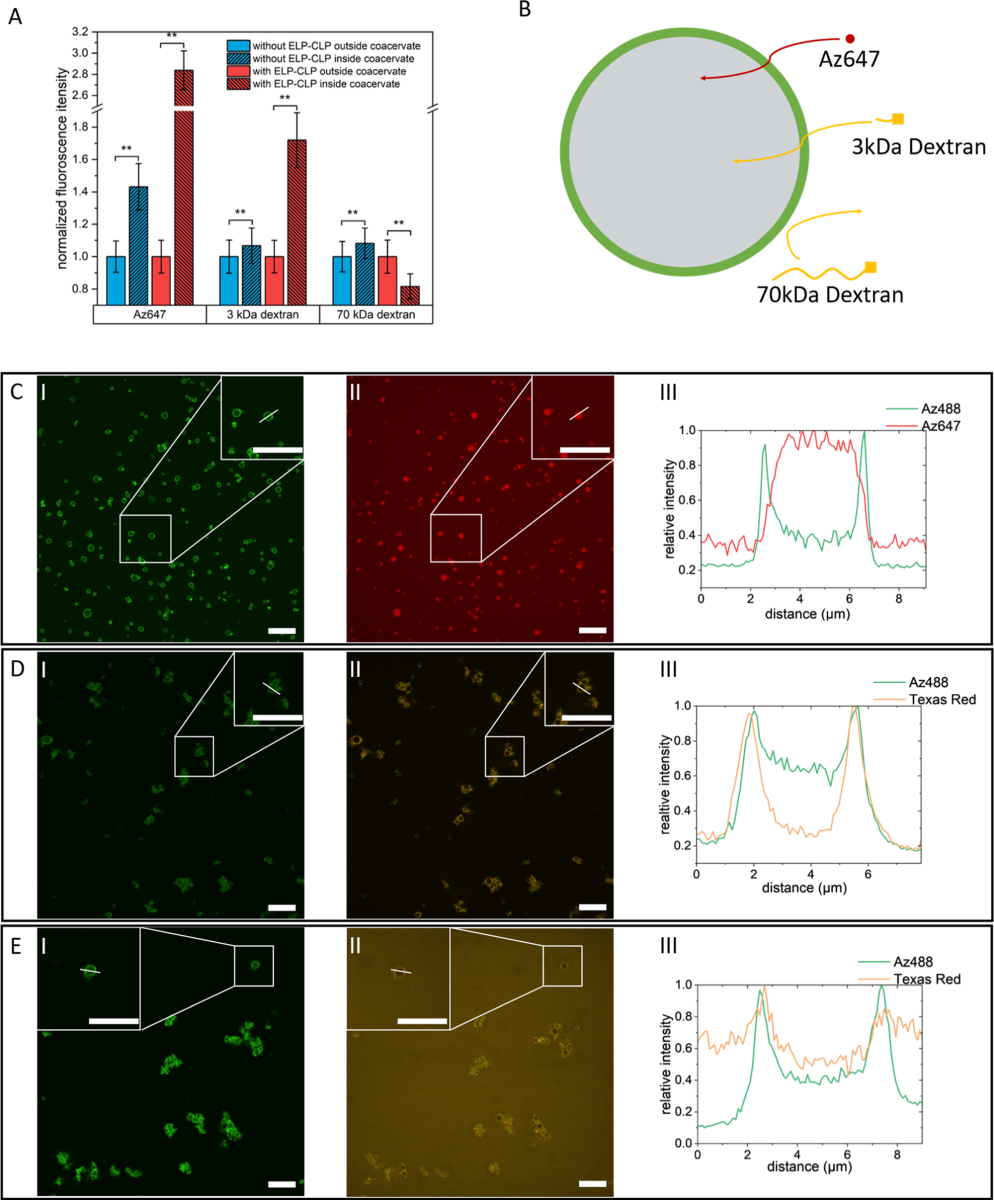
(A) Statistical analysis of fluorescence intensity distribution of selected molecules inside and outside coacervates. The fluorescence intensity was normalized to the solution background. **p < 0.01. (B) Scheme of diffusion test with selected molecules. (C) (I) Az488 (ELP-CLP label) and (II) Az647 (test molecule) channels of the confocal images collected after adding Az647 to coated coacervates. (III) Intensity map of the highlighted white line indicates the increased concentration of Az647 in coacervates. (D) (I) Az488 (ELP-CLP label) and (II) Texas Red (test molecule label) channels of the confocal images collected after adding Dextran (3K Da, Texas Red-labeled) to coated coacervates. (III) Intensity map of the highlighted white line indicates the increased concentration of Az647 in coacervates. (E) (I) Az488 (ELP-CLP label) and (II) Texas Red (test molecule label) channels of the confocal images collected after adding Dextran (70K Da, Texas Red-labeled) to coated coacervates. (III) Intensity map of the highlighted white line indicates the decreased concentration of Az647 in coacervates. All scale bars = 20 μm.

Our data indicate that electrostatic interactions of ELP-CLP vesicles with coacervates can be successfully leveraged to form a coating on the surface of preformed coacervates. The hydrophilic head (CLP domain, which assembled into a triple helix)^39^ performs as an oligomerization unit to increase the charge density at the end of the molecule, with the potential for improved stability over lipid-based membranes. The strategy of directly binding/forming amphiphilic bilayers on the surface of coacervates through electrostatic interactions has been successfully achieved with lipids^20,49^ and with fatty acids.^55^ In these previous studies, the components of the lipid coating have to be carefully tuned to improve the stability of the lipid bilayers while maintaining their coating properties. With ELP-CLP, the increased stability of ELP-CLP vesicles offers potential advantages with more diverse coating properties. In reports of polymeric materials, electrostatic approaches were less efficient due to the lower charge densities in polymeric systems and the distribution of charge along the polymer backbone. As a result, charged amphiphilic polymers (e.g., block copolymers comprising polystyrenesulfonate^56^ or polydimethyldiallyl ammonium^57^) commonly prefer to participate in the coacervation instead of coating the coacervate surface.^58,59^

The successful coating of ELP-CLP vesicles also provided a promising material system for the construction of functional coacervates with selective permeability. Penetration of molecules in lipid bilayer-coated coacervates or lipid vesicles was dependent on the integrity of the membrane. With a fully intact membrane, the diffusion of molecules is hindered except the diffusion of small nonpolar molecules, such as O_2_ and CO_2_,^20,60^ while a membrane with defects would allow the diffusion of most larger molecules.^49,55,61^ Thus, to provide selective diffusibility of different molecules with a lipid-based coating, very specific and controlled formulation processes to incorporate the membrane proteins in lipid bilayers are required.^62,63^ For example, coating of extracted cell membrane fragments is more commonly used when selective transport is required.^64^ Our ELP-CLP coating may thus provide a more facile synthetic approach to build coatings with MW-dependent permeability.

Precise characterization of the structure of the ELP-CLP coating of these coacervates will be required to fully understand the mechanism of the coating process and the ability to demonstrate a more precise selective permeability. In addition, the detailed characterization of other properties of the ELP-CLP coating, such as compatibility with different coacervate systems, maximum permeable molecule size, diffusion coefficient of different molecules, and long-term stability, will enable the modification of the reported coacervate systems and the design of new ELP-CLP coatings with desired properties.

### Conclusions

Here, we demonstrated applying assembled ELP-CLP vesicles to compartmentalize and stabilize a PLK/PLD coacervate system. Localization of the negatively charged ELP-CLP vesicles on the surface of the positively charged PLK/PLD coacervates was observed. Below a minimum coating concentration, ELP-CLP vesicles promoted the aggregation of the PLK/PLD coacervates, while with increased ELP-CLP concentration, an ELP-CLP coating formed gradually on the surface of the coacervates. The ELP-CLP coating increased the stability of coacervates: the coated coacervates were stable in solution for more than 1 week, whereas the uncoated coacervates redissolved in solution within 2 days. The coating of coacervates with ELP-CLPs was driven by electrostatic interactions, and thus tuning the coacervate surface charge or order of addition could be used to either dissuade or promote formation of the ELP-CLP coating and to either reduce or increase the colocalization of ELP-CLP inside the coacervates. The ELP-CLP coatings showed higher permeability for low MW molecules, whereas diffusion of high MW molecules across the coated coacervate surface was hindered, highlighting the possibility of applying ELP-CLPs as functional coatings to control molecule transfer in artificial organelles.

The charge interactions between ELP-CLP and coacervates could also be applied to other LLPS systems with surface charge to provide tunable surface modification that could enable controlled permeability and increased stability versus current artificial organelle systems composed of lipid membranes, which hinder the diffusion of most molecules and suffer from poor long-term stability. This work demonstrates an initial approach to controlling the coating process and coating functionality. Further investigation is still required to understand the coating structure and mechanism of the ELP-CLP coating process, the apparent diffusibility of different molecules within ELP-CLP coatings, and their diffusion mechanism.

## Materials and Methods

### Materials

All amino acids, resins, and activators for solid-phase peptide synthesis were purchased from ChemPep (Wellington, FL) and the CEM corporation (Matthews, NC) and used as received. Poly-l-lysine hydrochloride (PLK-100, MW = 16,000 Da) and poly-l-aspartic acid (PLD-100, MW = 11,500 Da) were purchased from Alamanda Polymers (Huntsville, AL). Fluorescent dye for ELP-CLP labeling (AF488 cadaverine, AF488 maleimide, and AF647 cadaverine) was purchased from Lumiprobe Corporation (Westminster, MD). Water for buffers was deionized and filtered by using either a ThermoFisher Barnstead NANOpure diamond water purifier or a Milli-Q Synergy water purification system. All other reagents were purchased and used as received from Sigma-Aldrich (St. Louis, MO) or Fisher Scientific (Hampton, NH) unless otherwise indicated.

### Peptide Synthesis and Purification

ELP and CLP polypeptides (sequences shown in Figure S1) were prepared using standard SPPS on a Liberty Blue automated microwave peptide synthesizer (CEM Corporation) at a 0.10 mmol scale. Fmoc-based protocols with a Rink amide resin (ChemPep) were used, yielding an amidated C-terminus after cleavage. The Fmoc was deprotected with 20% piperidine (Sigma) in dimethylformamide (DMF) (Fisher) at 75 °C for 3 min, along with 5 washes with DMF after deprotection. Subsequent coupling steps were performed at 90 °C for 5 min with 4 eq. of the appropriate protected amino acid (0.2 mM, ChemPep) or 4-azidobutyric acid (0.2 mM, Sigma), 4 equiv. ethyl (hydroxyimino)­cyanoacetate (Oxyma, 1 mM, CEM), and 4 eq. *N*,*N*′-diisopropylcarbodiimide (DIC, 1 mM, ChemImpex). To increase the yield of full-length peptides, all amino acids were double coupled. After synthesis, the peptides were washed with DMF and dichloromethane (DCM) (Fisher) 3 times and then cleaved in a 10 mL modified Reagent R cleavage solution (90% trifluoroacetic acid (Sigma), 5% DODT (Sigma), 3% thiolanisole (Sigma), and 2% anisole (Sigma)) by shaking at room temperature for 2 h. The cleaved peptide solutions were precipitated using cold ethyl ether and centrifuged at 4000 rpm for 5 min and washed with cold ethyl ether three times. The precipitates were dried overnight and redissolved in deionized 95% Milli-Q water and 5% ACN. The solutions were further purified via high-performance liquid chromatography (HPLC, Quaternary Gradient Module (Waters 2545), Waters Corporation, Milford, MA) using a reverse-phase BEH130 Prep C18 10 μm column (XBridge, Waters Corporation). Pure fractions of the peptides were combined and lyophilized. Their purity and molecular weight were confirmed via analytical ultrahigh performance mass spectrometry (UPLC-MS, Waters Xevo G2-S QTof, Waters Corporation).

### Preparation of Labeled ELP-CLP Vesicles

ELP (F_6_G’, G’ = propargyl glycine) and CLP (G_7_GG, G_7_GGCOOH, G_7_GC) polypeptides were first conjugated by copper­(I)-catalyzed azide–alkyne cycloaddition (CuAAC) reaction to synthesize the ELP-CLP conjugates. ELP (3 μmol), CLP (6 μmol), Cu­(II) sulfate (6 μmol), Tris­(3-hydroxypropyltriazolylmethyl)­amine (THPTA, 30 μmol), and (+)-sodium l-ascorbate (400 μmol) were dissolved in 7:3 DMSO/water (v/v) and incubated for 1 h with stirring at 70 °C. The conjugated ELP-CLPs (F_6_G_7_GG, F_6_G_7_GC, F_6_G_7_GGCOOH) were purified by HPLC at 70 °C. The self-assembly of F_6_G_7_GG was confirmed by DLS (Figure S8A), and the construct exhibited similar thermoresponsive behavior as previously observed.^65^ The functionalized ELP-CLP vesicles were assembled by mixing 9:1 F_6_G_7_GG/F_6_G_7_GC or F_6_G_7_GG/F_6_G_7_GGCOOH (v/v, all ELP-CLP were in a 2 mg/mL solution). The solution was incubated at 80 °C for 20 min to unfold the CLP domain and then cooled down to 37 °C and incubated overnight to allow the CLP refolding and self-assembly of ELP-CLP vesicles. To label the carboxyl group-functionalized ELP-CLP vesicles, the EDC/NHS reaction was carried out on the assembled vesicles. 0.4 mg of 1-ethyl-3-(3-(dimethylamino)­propyl)­carbodiimide (EDC, Thermo Scientific) and 1.1 mg of *N*-hydroxysulfosuccinimide (sulfo-NHS, Thermo Scientific) were added to 1 mL of carboxyl group-functionalized ELP-CLP vesicles and reacted at 37 °C on a rotating tube mixer operating at a rotation rate of 50 rpm for 15 min. A 10-fold excess of AF488-cadaverine was then added to the solution, and the mixture was reacted for another 2 h. For Cys-functionalized ELP-CLP vesicles, the thiol–maleimide reaction was carried out by mixing a 10-fold excess of AF488-maleimide with 1 mL of Cys-functionalized ELP-CLP vesicles followed by incubation at 37 °C for 15 min on a rotating tube mixer operating at 50 rpm. To purify the labeled coacervates, vesicles were collected via centrifugation (Eppendorf Centrifuge 5424, 21130 RCF for 5 min), and the supernatant was carefully removed via pipetting. A majority of the soluble dyes were removed with the supernatant, leaving the ELP-CLP vesicles in the spun-down fraction. To remove the remaining free dye, the ELP-CLP vesicles were resuspended in 1 mL of DI water by vortexing, centrifuged again, and the supernatant was removed. The process was repeated three times, and then the ELP-CLP vesicles were resuspended in 333 μL of water for further use.

### Formulation of ELP-CLP-Coated Coacervates

The coacervates were prepared by adding 500 μM PLK and 500 μM PLD stock solutions (total volume = 200 μL; volume ratios were varied depending on the desired properties of the coacervate) in a 300 μL pH 6.8 MES buffer and vortexed for 30 s to ensure thorough mixing. The different volume ratios of PLK and PLD resulted in a final PLD + PLK concentration of 200 μM, and the coacervate solution presented different surface charges with varied volume ratios, as shown in Table S1. The zeta potential of uncoated coacervates was confirmed by a ZetaSizer Nano Series instrument (Nano ZS, Malvern Panalytical, UK). The required ratio of PLK/PLD to prepare the coacervate with a positive or negative surface charge was confirmed for each batch of PLK and PLD stock solution before use (Table S1).

To prepare ELP-CLP-coated coacervates, different volumes of labeled ELP-CLP vesicles were carefully added to the coacervate solution and mixed by manual agitation, yielding 2:98, 5:95, and 10:90 ELP-CLP/coacervate (v/v) solutions. The ELP-CLP-coated coacervates were aged at room temperature for at least 30 min before imaging.

The influence of different formulation protocols on preparing the coated coacervate was carried out separately. To investigate the influence of the mixing process, a 10 vol % labeled ELP-CLP vesicle solution was added to the p-coacervate solution and vortexed for 30 s with a Vortex-Genie 2 (Scientific Industry, Inc., Bohemia, NY) at maximum speed. The influence of the order of addition was also carried out. Solutions containing ELP-CLP/PLK/PLD were added to pH 6.8 MES buffer in different orders. After each component was added, vortexing was applied with a Vortex-Genie 2 at a maximum speed to ensure thorough mixing. The coated coacervates with different formulation protocols were aged for approximately 30 min before imaging.

To test the permeability of ELP-CLP-coated coacervates, 0.02 mg/mL of AF647-cadaverine (Lumiprobe Corporation), 1 mg/mL of Texas Red-labeled dextran (MW = 3 kDa, Thermo Scientific), or 1 mg/mL of Texas Red-labeled dextran (MW = 70 kDa, Thermo Scientific) was added to the coacervate solution. The mixture was vortexed and aged for at least 1 h before imaging to establish the equilibrium between coacervates and solution.

### Confocal Imaging and Analysis

Coacervate samples were gently mixed by pipetting before the samples were prepared for confocal imaging. Confocal imaging was performed using an Andor Dragonfly 600 microscope (Oxford Instruments) with an Andor Zyla 4.2 PLUS sCMOS camera and a Leica Plan Apo 63× oil immersion TIRF objective (numerical aperture, 1.47). Labeled ELP-CLP vesicles were imaged with a 488 nm laser and a 521 bandpass (BP) filter. AF647 was imaged with a 638 nm laser and a 685 bandpass (BP) filter. Texas Red-labeled dextrans (3 and 70 kDa) were imaged with a 561 nm laser and a 594 bandpass (BP) filter.

The quantification of images was conducted with Fiji.^66^ Three 196 pixel × 196 pixel areas were selected manually, either inside or outside the coacervates. The fluorescence intensity inside and outside of the coacervates was calculated by determining the average fluorescence in these regions within the three sample areas. The intensity was normalized to the model molecule fluorescence intensity in solution for further data analysis. The fluorescence intensity on the surface of the coacervate was calculated by averaging the intensities of three 98-pixel lines placed on the high fluorescence intensity circle on the coacervate. The data were transferred to origin for visualization and statistical analysis. The statistical hypothesis tests to compare the statistical difference of intensities inside and outside coacervates were conducted by using the TwoSampletTest function in origin.

## Supplementary Material



## Notes

Synthesis and purification of ELP-CLP polypeptides (Figures S1–S7); characterization of ELP-CLP self-assembly before and after labeling (Figure S8); charge state of PLK/PLD coacervate without coating (Table S1); coated and uncoated coacervate size and morphology (Figure S9); stability test of uncoated coacervate (Figure S10); original image and summary of fluorescence intensity of diffused model molecules with uncoated coacervates (Figure S11 and Table S2); and summary of fluorescence intensity of diffused model molecules with uncoated coacervates (Table S3) (PDF)

## References

[1] Lee K. Y., Park S.-J., Lee K. A., Kim S.-H., Kim H., Meroz Y., Mahadevan L., Jung K.-H., Ahn T. K., Parker K. K., Shin K. (2018). Photosynthetic Artificial Organelles
Sustain and Control ATP-Dependent Reactions in a Protocellular System. Nat. Biotechnol..

[2] Otrin L., Kleineberg C., Caire da Silva L., Landfester K., Ivanov I., Wang M., Bednarz C., Sundmacher K., Vidaković-Koch T. (2019). Artificial Organelles for Energy
Regeneration. Adv. Biosyst..

[3] Silverman A. D., Karim A. S., Jewett M. C. (2020). Cell-Free Gene Expression: An Expanded
Repertoire of Applications. Nat. Rev. Genet..

[4] Hunt A. C., Rasor B. J., Seki K., Ekas H. M., Warfel K. F., Karim A. S., Jewett M. C. (2025). Cell-Free
Gene Expression: Methods
and Applications. Chem. Rev..

[5] Mukerabigwi J. F., Ge Z., Kataoka K. (2018). Therapeutic
Nanoreactors as In Vivo Nanoplatforms for
Cancer Therapy. Chem—Eur. J..

[6] Giessen T. W., Silver P. A. (2016). A Catalytic Nanoreactor
Based on in Vivo Encapsulation
of Multiple Enzymes in an Engineered Protein Nanocompartment. ChemBioChem.

[7] Oerlemans R. A. J. F., Timmermans S. B. P. E., van Hest J. C. M. (2021). Artificial Organelles:
Towards Adding or Restoring Intracellular Activity. ChemBioChem.

[8] Godoy-Gallardo M., York-Duran M. J., Hosta-Rigau L. (2018). Recent Progress in Micro/Nanoreactors
toward the Creation of Artificial Organelles. Adv. Healthc. Mater..

[9] Refaat A., del Rosal B., Palasubramaniam J., Pietersz G., Wang X., Moulton S. E., Peter K. (2021). Near-Infrared Light-Responsive Liposomes
for Protein Delivery: Towards Bleeding-Free Photothermally-Assisted
Thrombolysis. J. Controlled Release.

[10] Zhao H., Ibarboure E., Ibrahimova V., Xiao Y., Garanger E., Lecommandoux S. (2021). Spatiotemporal
Dynamic Assembly/Disassembly of Organelle-Mimics
Based on Intrinsically Disordered Protein-Polymer Conjugates. Adv. Sci..

[11] Cao S., Ivanov T., Heuer J., Ferguson C. T. J., Landfester K., Caire da Silva L. (2024). Dipeptide
Coacervates as Artificial Membraneless Organelles
for Bioorthogonal Catalysis. Nat. Commun..

[12] Cook A. B., Novosedlik S., van Hest J. C. M. (2023). Complex Coacervate Materials as Artificial
Cells. Acc. Mater. Res..

[13] Zhang Y., Chen Y., Yang X., He X., Li M., Liu S., Wang K., Liu J., Mann S. (2021). Giant Coacervate Vesicles
As an Integrated Approach to Cytomimetic Modeling. J. Am. Chem. Soc..

[14] Banani S. F., Lee H. O., Hyman A. A., Rosen M. K. (2017). Biomolecular Condensates:
Organizers of Cellular Biochemistry. Nat. Rev.
Mol. Cell Biol..

[15] Wei M., Wang X., Qiao Y. (2024). Multiphase Coacervates: Mimicking
Complex Cellular Structures through Liquid–Liquid Phase Separation. Chem. Commun..

[16] Nott T. J., Craggs T. D., Baldwin A. J. (2016). Membraneless Organelles Can Melt
Nucleic Acid Duplexes and Act as Biomolecular Filters. Nat. Chem..

[17] Capasso
Palmiero U., Paganini C., Kopp M. R. G., Linsenmeier M., Küffner A. M., Arosio P. (2022). Programmable Zwitterionic Droplets
as Biomolecular Sorters and Model of Membraneless Organelles. Adv. Mater..

[18] Song S., Llopis-Lorente A., Mason A. F., Abdelmohsen L. K. E.
A., van Hest J. C. M. (2022). Confined
Motion: Motility of Active
Microparticles in Cell-Sized Lipid Vesicles. J. Am. Chem. Soc..

[19] Deshpande S., Brandenburg F., Lau A., Last M. G. F., Spoelstra W. K., Reese L., Wunnava S., Dogterom M., Dekker C. (2019). Spatiotemporal
Control of Coacervate Formation within Liposomes. Nat. Commun..

[20] Pir
Cakmak F., Marianelli A. M., Keating C. D. (2021). Phospholipid Membrane
Formation Templated by Coacervate Droplets. Langmuir.

[21] Paganini C., Capasso Palmiero U., Picciotto S., Molinelli A., Porello I., Adamo G., Manno M., Bongiovanni A., Arosio P. (2023). High-Yield Separation of Extracellular Vesicles Using
Programmable Zwitterionic Coacervates. Small.

[22] Akter A., Zhan W. (2025). Janus Liposomes: Exploring Liquid–Liquid
Phase-Separating
Lipid Systems Alternative to DOPC/DPPC/Cholesterol. Langmuir.

[23] Li Q., Song Q., Guo W., Cao Y., Cui X., Chen D., Shum H. C. (2023). Synthetic Membraneless Droplets for
Synaptic-Like Clustering of Lipid Vesicles. Angew. Chem..

[24] Wang T., Bai J., Jiang X., Nienhaus G. U. (2012). Cellular Uptake of Nanoparticles
by Membrane Penetration: A Study Combining Confocal Microscopy with
FTIR Spectroelectrochemistry. ACS Nano.

[25] Lu T., Javed S., Bonfio C., Spruijt E. (2023). Interfacing Coacervates
with Membranes: From Artificial Organelles and Hybrid Protocells to
Intracellular Delivery. Small Methods.

[26] Hashemzadeh H., Javadi H., Darvishi M. H. (2020). Study of Structural
Stability and
Formation Mechanisms in DSPC and DPSM Liposomes: A Coarse-Grained
Molecular Dynamics Simulation. Sci. Rep..

[27] Sułkowski W. W., Pentak D., Nowak K., Sułkowska A. (2005). The Influence
of Temperature, Cholesterol Content and pH on Liposome Stability. J. Mol. Struct..

[28] Shinoda W. (2016). Permeability
across Lipid Membranes. Biochim. Biophys. Acta
Biomembr..

[29] Fujii S., Matsuura T., Sunami T., Nishikawa T., Kazuta Y., Yomo T. (2014). Liposome Display for in Vitro Selection
and Evolution of Membrane Proteins. Nat. Protoc..

[30] Gu Y., Wang R., Chen P., Li S., Chai X., Chen C., Liu Y., Cao Y., Lv D., Hong Z., Zhu Z., Chai Y., Yuan Y., Chen X. (2022). *In Situ* Synthesis and Unidirectional Insertion of
Membrane Proteins in Liposome-Immobilized Silica Stationary Phase
for Rapid Preparation of Microaffinity Chromatography. Acta Pharm. Sin. B.

[31] Ji Y., Mu W., Wu H., Qiao Y. (2021). Directing Transition
of Synthetic
Protocell Models via Physicochemical Cues-Triggered Interfacial Dynamic
Covalent Chemistry. Adv. Sci..

[32] Tonga G. Y., Jeong Y., Duncan B., Mizuhara T., Mout R., Das R., Kim S. T., Yeh Y.-C., Yan B., Hou S., Rotello V. M. (2015). Supramolecular Regulation of Bioorthogonal
Catalysis
in Cells Using Nanoparticle-Embedded Transition Metal Catalysts. Nat. Chem..

[33] Yusop R. M., Unciti-Broceta A., Johansson E. M. V., Sánchez-Martín R. M., Bradley M. (2011). Palladium-Mediated Intracellular Chemistry. Nat. Chem..

[34] Miller M. A., Mikula H., Luthria G., Li R., Kronister S., Prytyskach M., Kohler R. H., Mitchison T., Weissleder R. (2018). Modular Nanoparticulate Prodrug Design Enables Efficient
Treatment of Solid Tumors Using Bioorthogonal Activation. ACS Nano.

[35] Bai Y., Feng X., Xing H., Xu Y., Kim B. K., Baig N., Zhou T., Gewirth A. A., Lu Y., Oldfield E., Zimmerman S. C. (2016). A Highly Efficient Single-Chain Metal–Organic
Nanoparticle Catalyst for Alkyne–Azide “Click”
Reactions in Water and in Cells. J. Am. Chem.
Soc..

[36] Van
Oppen L. M. P. E., Abdelmohsen L. K. E.
A., Van Emst-de
Vries S. E., Welzen P. L. W., Wilson D. A., Smeitink J. A. M., Koopman W. J. H., Brock R., Willems P. H. G. M., Williams D. S., Van Hest J. C. M. (2018). Biodegradable Synthetic Organelles
Demonstrate ROS Shielding in Human-Complex-I-Deficient Fibroblasts. ACS Cent. Sci..

[37] Maffeis V., Heuberger L., Nikoletić A., Schoenenberger C.-A., Palivan C. G. (2024). Synthetic Cells Revisited: Artificial
Cell Construction
Using Polymeric Building Blocks. Adv. Sci..

[38] Ji Y., Qiao Y. (2024). Tuning Interfacial Fluidity and Colloidal Stability
of Membranized
Coacervate Protocells. Commun. Chem..

[39] Luo T., Kiick K. L. (2015). Noncovalent Modulation
of the Inverse Temperature Transition
and Self-Assembly of Elastin-b-Collagen-like Peptide Bioconjugates. J. Am. Chem. Soc..

[40] Taylor P. A., Huang H., Kiick K. L., Jayaraman A. (2020). Placement
of Tyrosine Residues as a Design Element for Tuning the Phase Transition
of Elastin-Peptide-Containing Conjugates: Experiments and Simulations. Mol. Syst. Des. Eng..

[41] Qin J., Sloppy J. D., Kiick K. L. (2020). Fine Structural Tuning of the Assembly
of ECM Peptide Conjugates via Slight Sequence Modifications. Sci. Adv..

[42] Wang B., Xie W., Zhang T., Pochan D. J., Saven J. G., Kiick K. L. (2025). Architectural
Control of Rod-Coil Block Polypeptide Thermoresponsive Self-Assembly
via de Novo Design of Coiled-Coil Orientation. J. Mater. Chem. B.

[43] Hwang J., Huang H., Sullivan M. O., Kiick K. L. (2023). Controlled Delivery
of Vancomycin from Collagen-Tethered Peptide Vehicles for the Treatment
of Wound Infections. Mol. Pharmaceutics.

[44] Luo T., David M. A., Dunshee L. C., Scott R. A., Urello M. A., Price C., Kiick K. L. (2017). Thermoresponsive
Elastin-b-Collagen-Like
Peptide Bioconjugate Nanovesicles for Targeted Drug Delivery to Collagen-Containing
Matrices. Biomacromolecules.

[45] Peydayesh M., Kistler S., Zhou J., Lutz-Bueno V., Victorelli F. D., Meneguin A. B., Spósito L., Bauab T. M., Chorilli M., Mezzenga R. (2023). Amyloid-Polysaccharide
Interfacial Coacervates as Therapeutic Materials. Nat. Commun..

[46] Krounbi L., Hedderick K., Eyal Z., Aram L., Shimoni E., Estroff L. A., Gal A. (2021). Surface-Induced Coacervation Facilitates
Localized Precipitation of Mineral Precursors from Dilute Solutions. Chem. Mater..

[47] Wilson C. G., Sisco P. N., Gadala-Maria F. A., Murphy C. J., Goldsmith E. C. (2009). Polyelectrolyte-Coated
Gold Nanorods and Their Interactions with Type I Collagen. Biomaterials.

[48] Yi L., Guo R., Yin Z., Fu Z., Guo J., Yin W., Fu Z., Huang C., Zou Z. (2025). Tuning the Intrafibrillar Collagen
Mineralization Rate and Mechanical Properties Through Polyelectrolyte-Controlled
Formation and Crystallization of Amorphous Precursor. Small.

[49] Pir
Cakmak F., Grigas A. T., Keating C. D. (2019). Lipid Vesicle-Coated
Complex Coacervates. Langmuir.

[50] Gao N., Mann S. (2023). Membranized Coacervate
Microdroplets: From Versatile Protocell Models
to Cytomimetic Materials. Acc. Chem. Res..

[51] Zhang Y., Wang Z., Li M., Xu C., Gao N., Yin Z., Wang K., Mann S., Liu J. (2023). Osmotic-Induced Reconfiguration
and Activation in Membranized Coacervate-Based Protocells. J. Am. Chem. Soc..

[52] Bygdeman S., Tangen O. (1975). The Effect of Dextran on Collagen-Induced Platelet
Aggregation in Vitro. Thromb. Res..

[53] Banerjee S., Szepes M., Dibbert N., Rios-Camacho J.-C., Kirschning A., Gruh I., Dräger G. (2021). Dextran-Based
Scaffolds for *in-Situ* Hydrogelation: Use for next
Generation of Bioartificial Cardiac Tissues. Carbohydr. Polym..

[54] Zhang X., Yang Y., Yao J., Shao Z., Chen X. (2014). Strong Collagen
Hydrogels by Oxidized Dextran Modification. ACS Sustain. Chem. Eng..

[55] Dora
Tang T.-Y., Rohaida Che Hak C., Thompson A. J., Kuimova M. K., Williams D. S., Perriman A. W., Mann S. (2014). Fatty Acid Membrane
Assembly on Coacervate Microdroplets as a Step towards a Hybrid Protocell
Model. Nat. Chem..

[56] Späth F., Donau C., Bergmann A. M., Kränzlein M., Synatschke C. V., Rieger B., Boekhoven J. (2021). Molecular
Design of Chemically Fueled Peptide–Polyelectrolyte Coacervate-Based
Assemblies. J. Am. Chem. Soc..

[57] Zhuang M., Zhang Y., Zhou S., Zhang Y., Wang K., Nie J., Liu J. (2019). Uricase-Containing
Coacervate Microdroplets as Enzyme
Active Membrane-Free Protocells for Detoxification of Uric Acid in
Serum. Chem. Commun..

[58] Sathyavageeswaran A., Bonesso Sabadini J., Perry S. L. (2024). Self-Assembling Polypeptides in Complex
Coacervation. Acc. Chem. Res..

[59] van
Westerveld L., Pelras T., Hofman A. H., Loos K., Kamperman M., Es Sayed J. (2024). Effect of Polyelectrolyte Charge
Density on the Linear Viscoelastic Behavior and Processing of Complex
Coacervate Adhesives. Macromolecules.

[60] Gao N., Mann S. (2023). Membranized Coacervate
Microdroplets: From Versatile Protocell Models
to Cytomimetic Materials. Acc. Chem. Res..

[61] Son J., Jung Y. (2022). Lipid Coated Protein
Condensates as Stable Protocells with Selective
Uptake Abilities for Biomolecules. Chem. Sci..

[62] Meyer C., Arizzi A., Henson T., Aviran S., Longo M. L., Wang A., Tan C. (2025). Designer Artificial Environments
for Membrane Protein Synthesis. Nat. Commun..

[63] Gaffney K. A., Guo R., Bridges M. D., Muhammednazaar S., Chen D., Kim M., Yang Z., Schilmiller A. L., Faruk N. F., Peng X., Jones A. D., Kim K. H., Sun L., Hubbell W. L., Sosnick T. R., Hong H. (2022). Lipid Bilayer Induces Contraction
of the Denatured State Ensemble of a Helical-Bundle Membrane Protein. Proc. Natl. Acad. Sci. U. S. A..

[64] Tan J., Zhu C., Li L., Wang J., Xia X.-H., Wang C. (2024). Engineering
Cell Membranes: From Extraction Strategies to Emerging Biosensing
Applications. Anal. Chem..

[65] Dunshee L. C., Sullivan M. O., Kiick K. L. (2020). Manipulation
of the Dually Thermoresponsive
Behavior of Peptide-based Vesicles through Modification of Collagen-like
Peptide Domains. Bioeng. Transl. Med..

[66] Fiji . ImageJ Wiki. https://imagej.github.io/software/fiji/index (accessed August 17, 2025).

