# Supplementary material for: Construction of Peptide Amphiphile-Coated Coacervates with Selective Permeability
Source: ACS Biomater Sci Eng. 2026 Feb 17;12(3):1856–67. doi: 10.1021/acsbiomaterials.5c02101 (PMC12976995; doi:10.1021/acsbiomaterials.5c02101)
Supplement: Supplementary file 1 [file ab5c02101_si_001.pdf]

Supporting Information

Construction of peptide amphiphile-coated coacervates with selective permeability

Bin Wang[a], Kristi L. Kiick* [a,b], and Millicent O. Sullivan* [b,c]

[a.] Department of Materials Science and Engineering, University of Delaware, Newark, DE, USA.
[b.] Department of Biomedical Engineering, University of Delaware, Newark, DE, USA
[c.] Department of Chemical and Biomolecular Engineering, University of Delaware, Newark, DE, USA
[*] Corresponding author. Email: msulliva@udel.edu, kiick@udel.edu

Synthesis and Purification of ELP-CLP polypeptides (Fig. S1 to S7):

$(VPGFG)_6$ with amide C-terminus, abbreviated as F_6G'
NH_2-VPGFGVPGFGVPGFGVPGFGVPGFGVPGFGG'-$CONH_2$

$(GPO)_7GG$ with amide C-terminus, abbreviated as G_7GG
(4-ABA)-GPOGPOGPOGPOGPOGPOGG-$CONH_2$

$(GPO)_7GC$ with amide C-terminus, abbreviated as G_7GC
(4-ABA)-GPOGPOGPOGPOGPOGPOGC-$CONH_2$

$(GPO)_7GG$ with carboxyl C-terminus, abbreviated as $G_7GGCOOH$
(4-ABA)-GPOGPOGPOGPOGPOGPO-COOH

G′: propargyl glycine
4-ABA: 4-azidobutanoic acid
O: hydroxyproline

Fig. S1. Detailed sequence of ELP and CLP peptides.

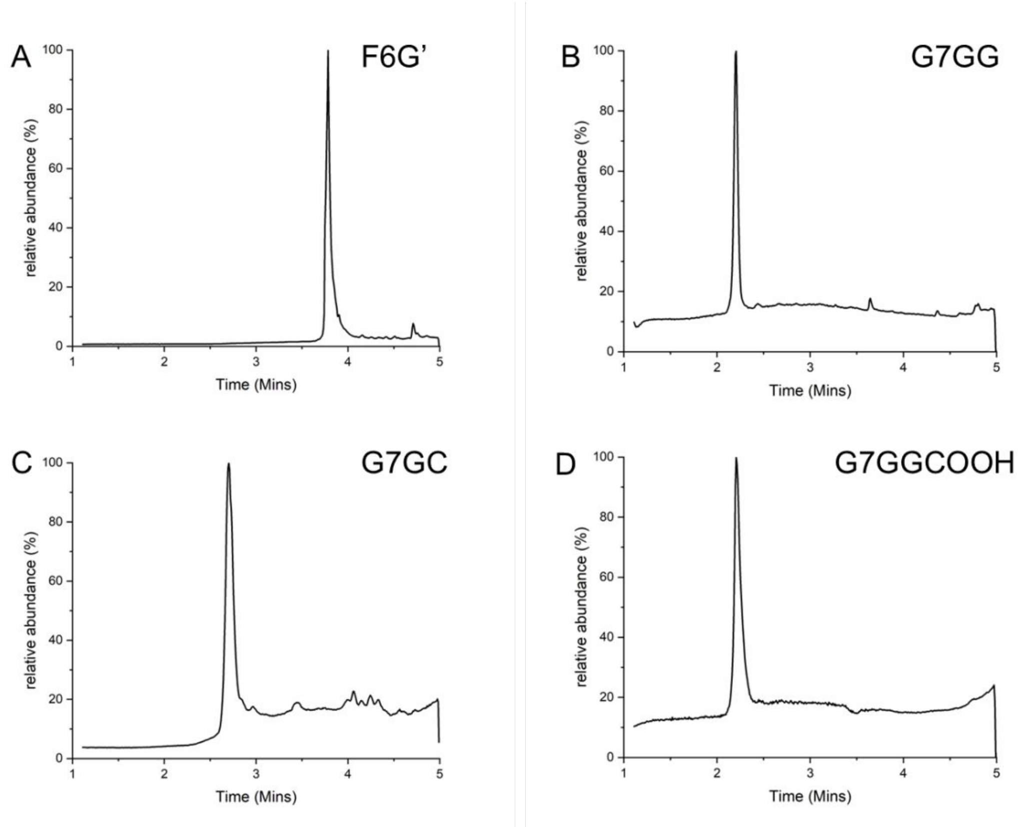

Fig. S2. UPLC of synthesized ELP and CLP sequences. The HPLC-purified lyophilized peptides were redissolved in DI water at 50 μM and the UPLC measurement was carried out in a Waters ACQUITY Ultra-Performance Liquid Chromatography (UPLC) system.

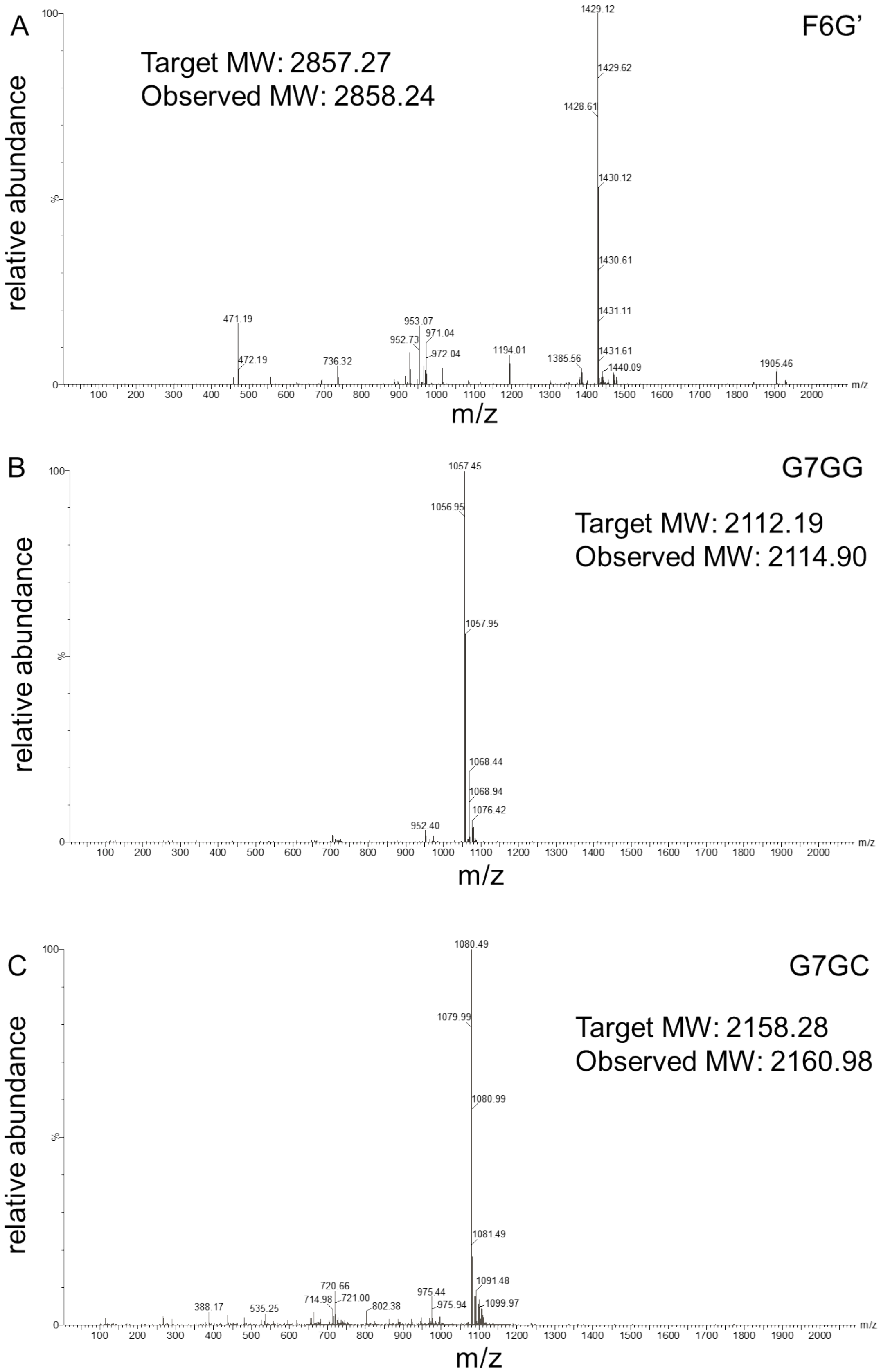
A
F6G’
Target MW: 2857.27
Observed MW: 2858.24
relative abundance
m/z
1429.12
1429.62
1428.61
1430.12
1430.61
1431.11
1431.61
1440.09
471.19
472.19
736.32
953.07
952.73
971.04
972.04
1194.01
1385.56
1905.46
B
G7GG
Target MW: 2112.19
Observed MW: 2114.90
relative abundance
m/z
1057.45
1056.95
1057.95
1068.44
1068.94
1076.42
952.40
C
G7GC
Target MW: 2158.28
Observed MW: 2160.98
relative abundance
m/z
1080.49
1079.99
1080.99
1081.49
1091.48
1099.97
975.44
975.94
720.66
714.98
721.00
802.38
388.17
535.25

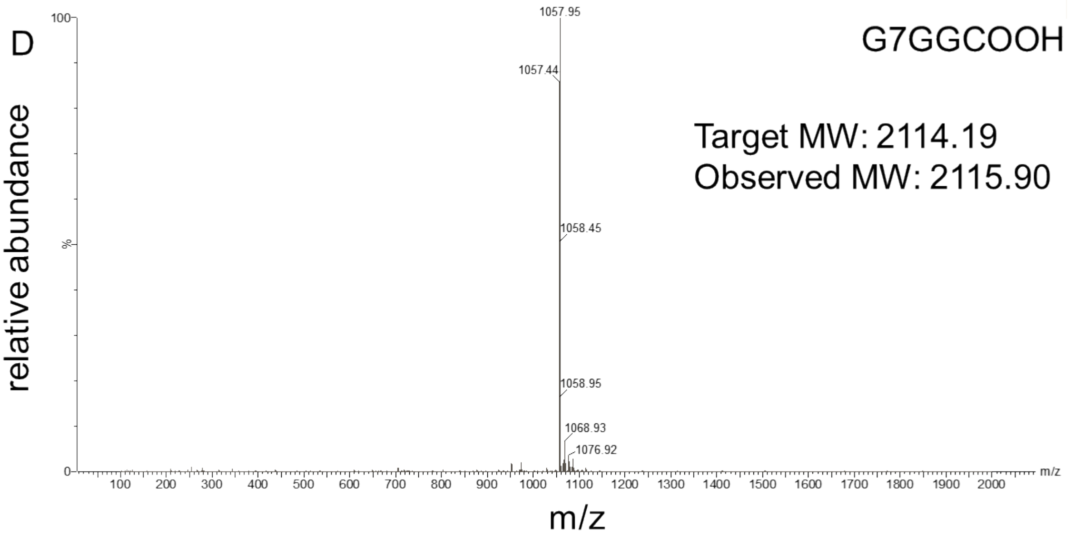

Fig. S3. Mass spectra of individual ELP and CLP peptides.

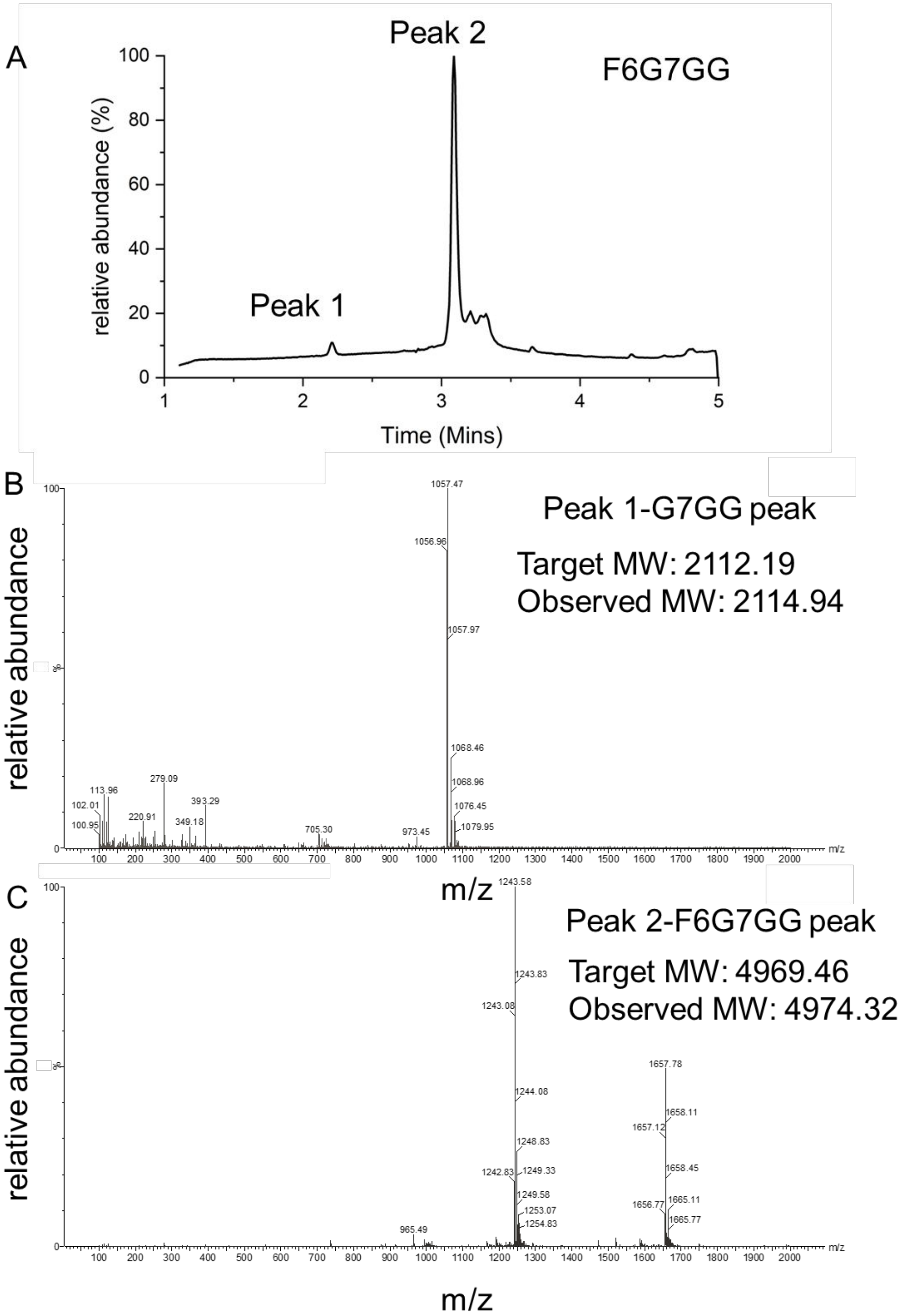

Fig. S4. UPLC and QTOF MS data of conjugated F_6G_7GG. Two peaks, representing CLP and ELP-CLP, were observed in UPLC due to the formation of a hybrid CLP triple helix from unconjugated CLP and conjugated ELP-CLP during purification process. A similar CLP peak was observed in our

previous ELP-CLP polypeptides but the presence of unconjugated CLP did not show significant influence on ELP-CLP vesicle assembly.[1]

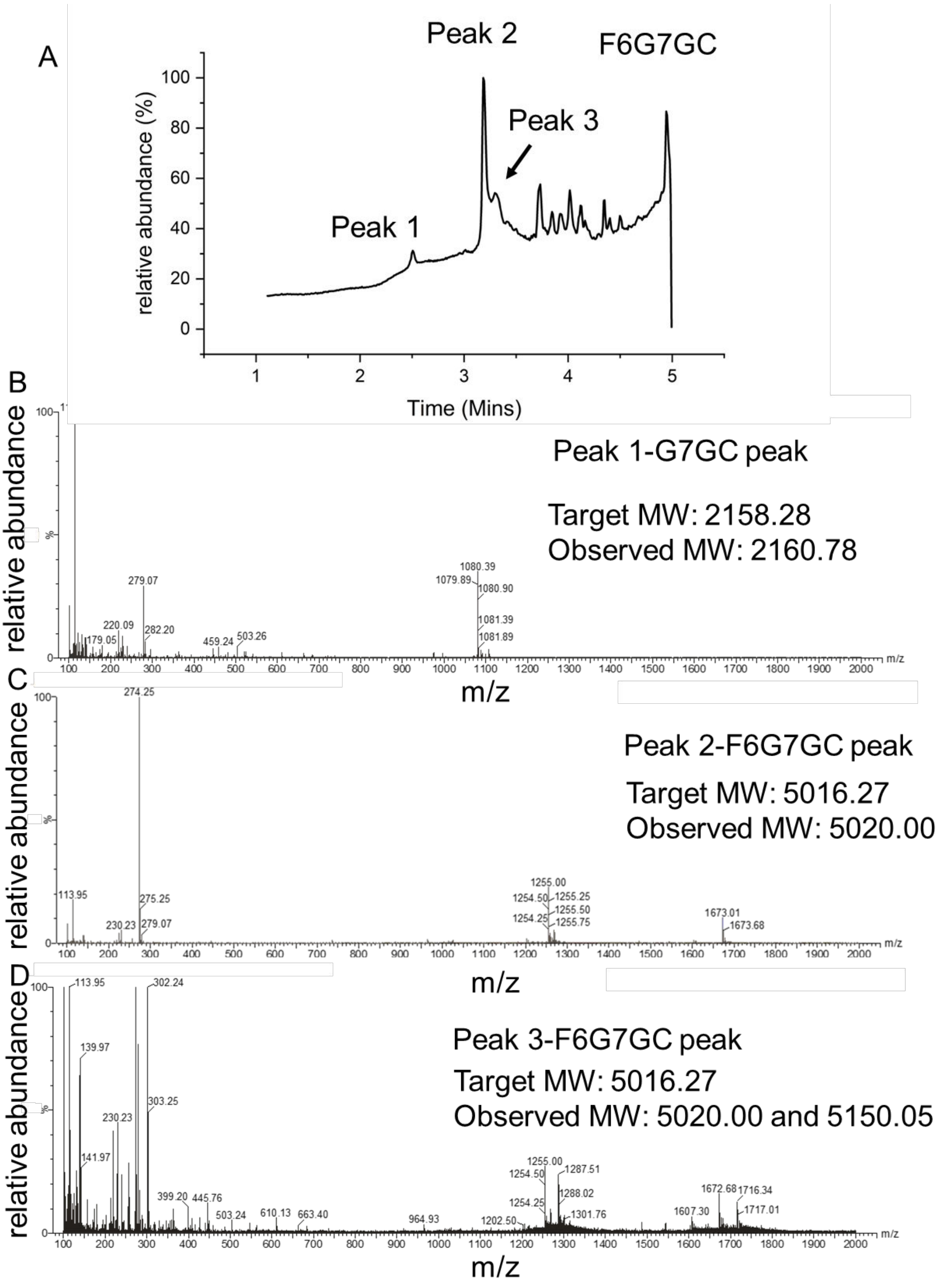

Fig. S5. UPLC and QTOF MS data of conjugated F_6G_7GC. The first and second peaks, representing CLP and ELP-CLP, were observed in UPLC due to the formation of hybrid CLP triple helix from unconjugated CLP and conjugated ELP-CLP during the purification process. Peak 3 is the ELP-CLP trimer peak. The peaks after 3.5 mins in UPLC were attributable to impurities in the UPLC column.

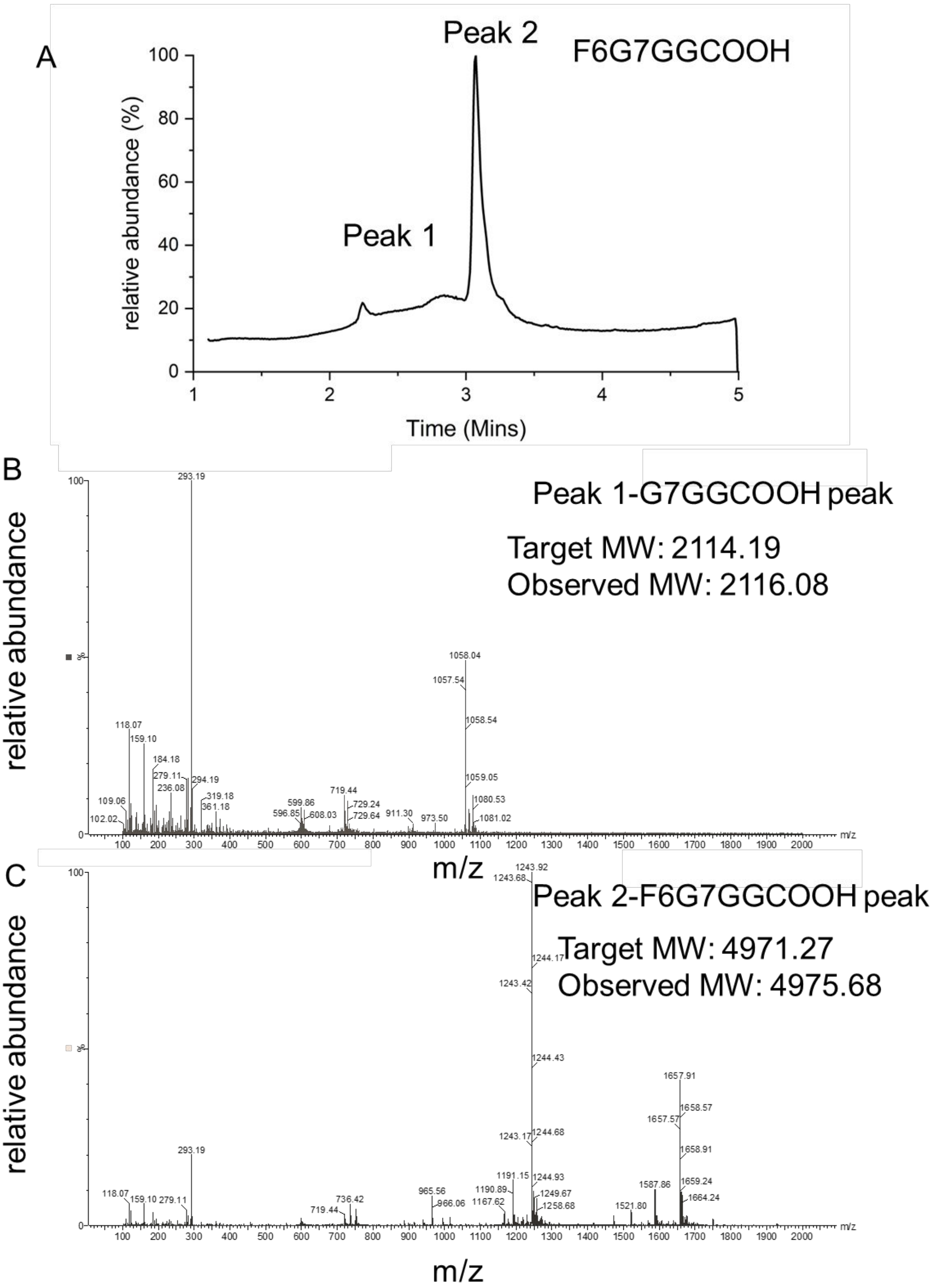

Fig. S6. UPLC and QTOF MS data of conjugated F_6G_7GGCOOH. Two peaks, representing CLP and ELP-CLP, were observed in UPLC due to the formation of a hybrid CLP triple helix from unconjugated CLP and conjugated ELP-CLP during purification process.

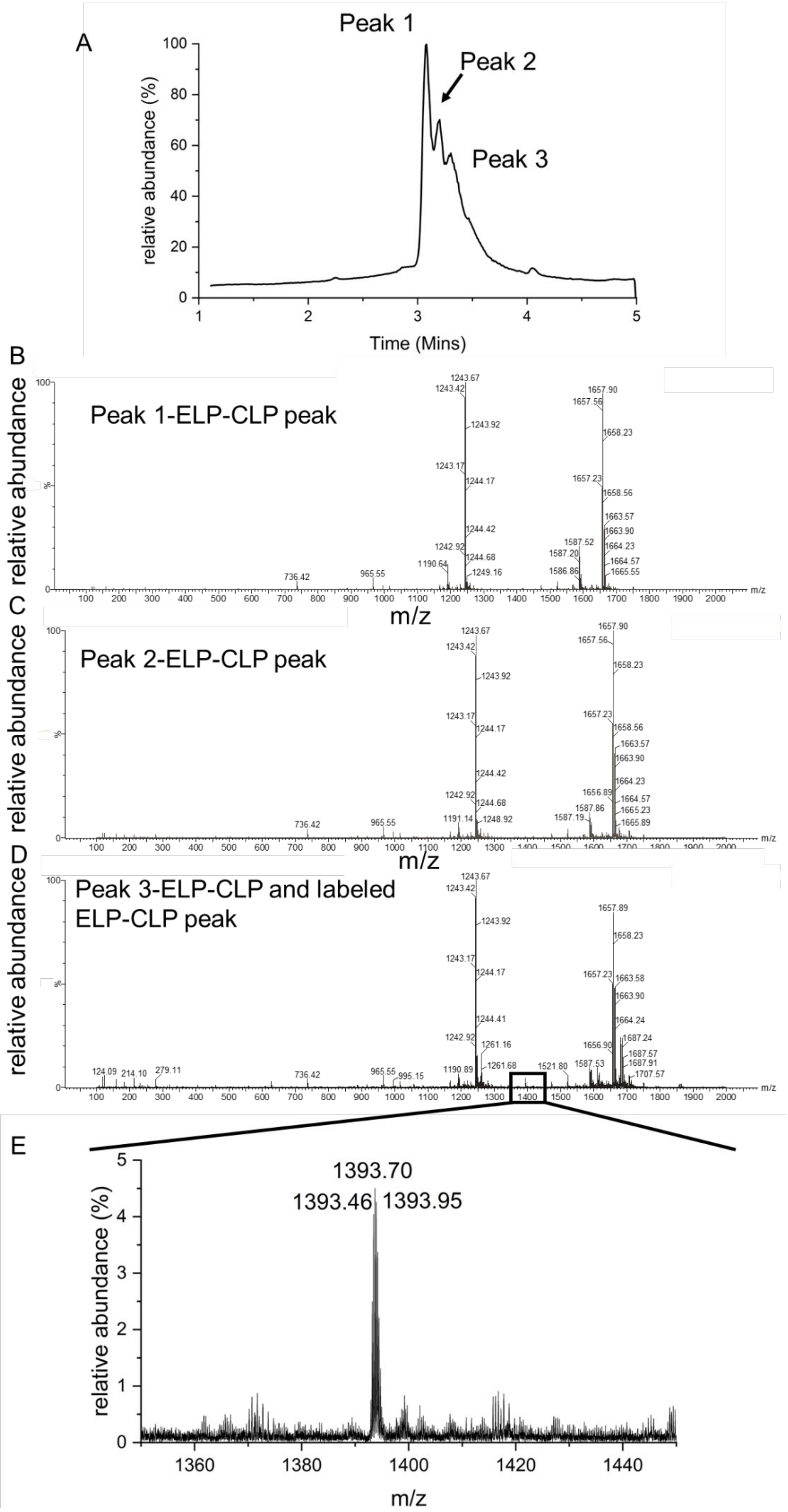
A
Peak 1
Peak 2
Peak 3
relative abundance (%)
Time (Mins)
B
Peak 1-ELP-CLP peak
relative abundance
m/z
C
Peak 2-ELP-CLP peak
relative abundance
m/z
D
Peak 3-ELP-CLP and labeled ELP-CLP peak
relative abundance
E
1393.70
1393.46
1393.95
relative abundance (%)
m/z

Fig. S7. UPLC and QTOF MS data of Az488 labeled ELP-CLP vesicles. The similar MW of $F_6G_7GGCOOH$ (target MW=4971.27 Da) and F_6G_7GG (target MW=4969.46 Da) prevented the separation of these two products under the MS conditions available for characterization (observed MW=4974.68 Da). The Az488 labeled $F_6G_7GGCOOH$ (target MW= 5571.90 Da, observed MW=5574.80 Da) was also observed. However, the low signal strength of Az488 labeled $F_6G_7GGCOOH$ (5% relative intensity in peak 3) suggests that the $F_6G_7GGCOOH$ (10% of total ELP-CLP) was not fully labeled. The unmodified carboxyl group provided negative charge in ELP-CLP vesicles.

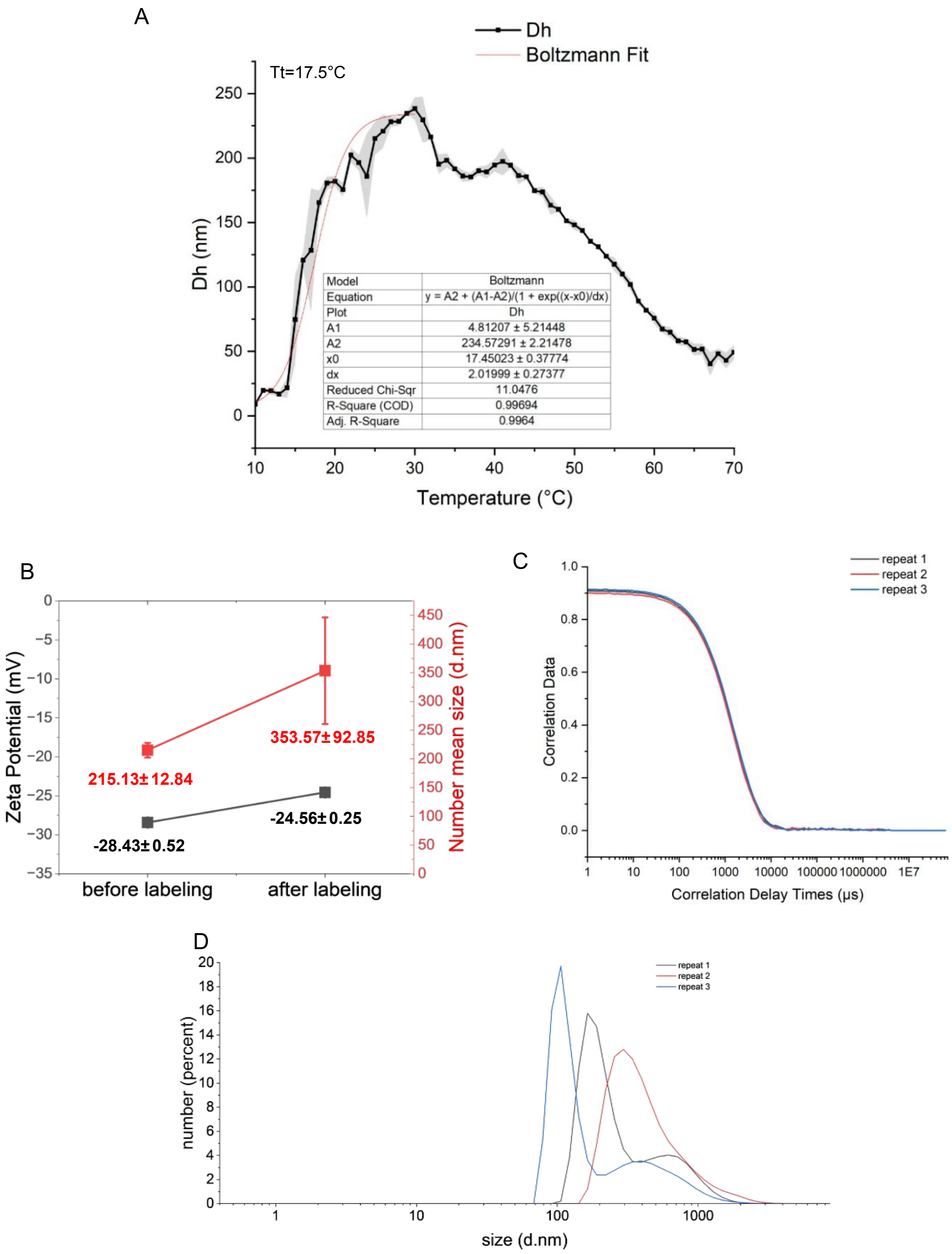

Fig. S8. A) Hydrodynamic diameter (Dh) of ELP-CLP vesicles at different temperatures, B) the surface charge and size change of ELP-CLP vesicles after labeling, and C) correlation data and D) size distribution of ELP-CLP vesicles after labeling. The thermoresponsiveness and self-assembly properties observed for the labeled ELP-CLP vesicles were similar to what was observed previously for the uncharged F6G7GG.[2] After labeling, the ZP was slightly increased, indicating the reaction of the carboxylate with the dye. The very slight change in surface charge indicated the labeling

reaction was not highly efficient. However, the independent characterization with confocal microscopy showed the fluorescence was sufficient for detection. The observed increase in diameter of the labeled vesicles is attributed to the aggregation of the ELP-CLP vesicles during the labeling process. The aggregation also caused the signals at high correlation delay times. Due to the formation of aggregates during labeling, the number mean fitting of correlation data varied from repeating experiments.

PLD (μM)	PLK (μM)	Zeta potential (mV)	Derived count rate (kcps)	comments
113	87	-34.97±1.54	34960.57±5858.82	applied as n-coacervate
110	90	-29.40±1.66	24644.03±2232.21	
104	96	-9.90±1.08	49104.47±11884.9	
100	100	11.97±1.38	61695.13±9015.044	
98	102	36.33±3.31	46188.20±9226.67	applied as p-coacervate
96	104	42.80±3.98	21443.93±1238.46	

Table S1. Table of coacervate charge with different PLK/PLD molar ratios. Concentration of PLD/PLK is shown as the final concentration in the coacervate solution. The molar ratio of PLK/PLD to prepare p-/n-coacervate was different for each batch of PLK and PLD stock solution. The molar ratio of PLK/PLD required to prepare p-/n-coacervate was tested through ZP for each batch of PLK and PLD stock solution before use to produce coacervates with similar surface charges.

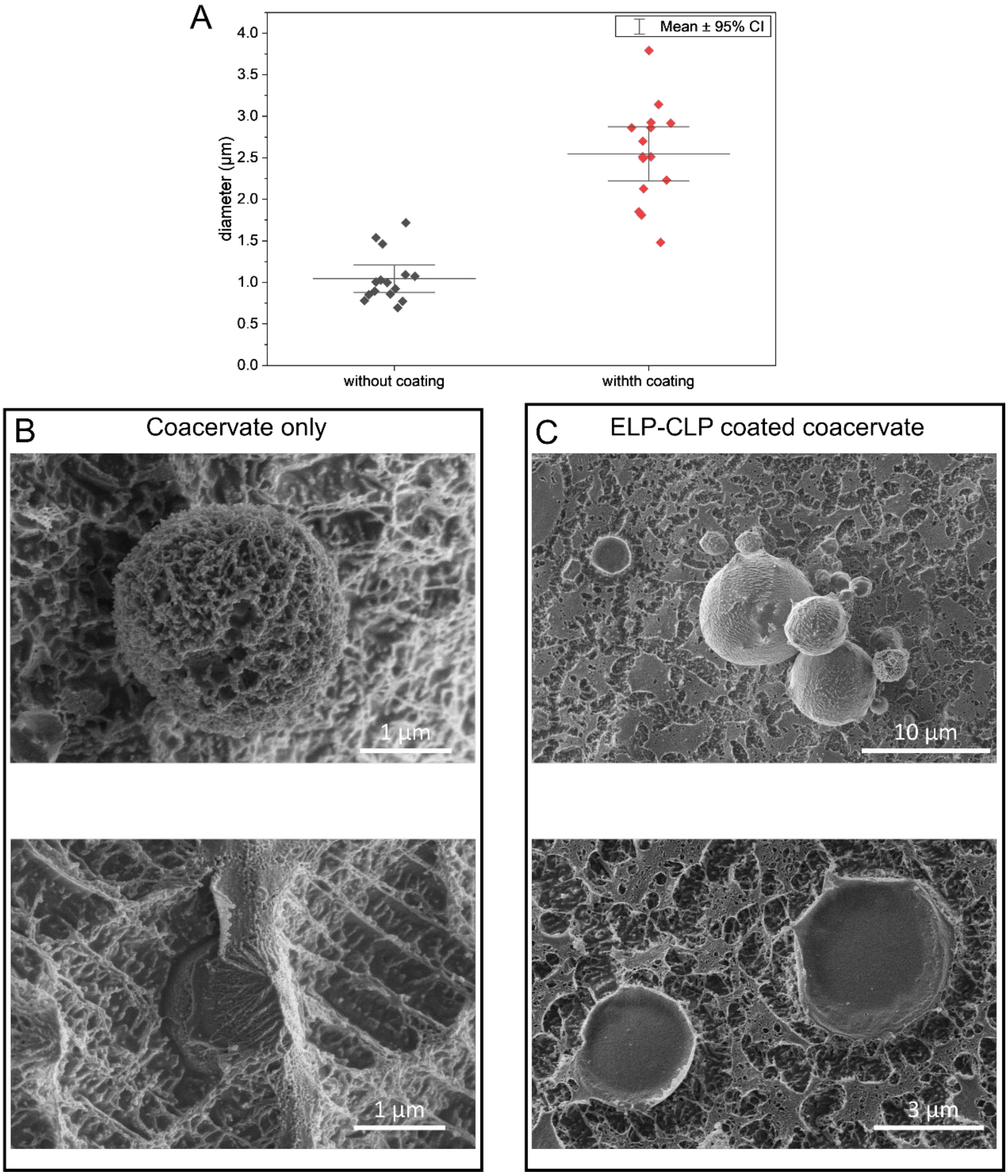

Fig. S9. A) Statistical analysis of coacervate without and with ELP-CLP coating. Cryo SEM results for B) p-coacervate and C) ELP-CLP-coated coacervate that show full coacervate particles and the cross-section of coacervates. Cryo SEM samples of p-coacervates and coated coacervate (90 p-coacervate : 10 ELP-CLP) were prepared using Leica EM ICE High Pressure Freezer, and the microtome SEM images were collected by Apreo VolumeScop Scanning Electron Microscope with integrated ultramicrotome.

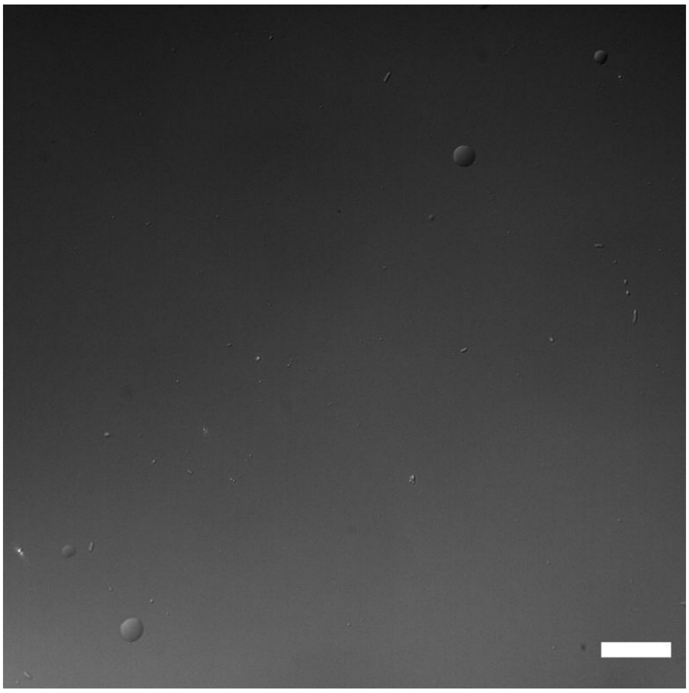

Fig. S10. DIC image of p-coacervate after aging at room temperature for 1 day. The same sample from Figure 2A was kept at room temperature for 24 hours. The turbid coacervate solution turned clear, and very few coacervates could be observed in the DIC channel of confocal microscopy after aging. Scale bar=20 µm

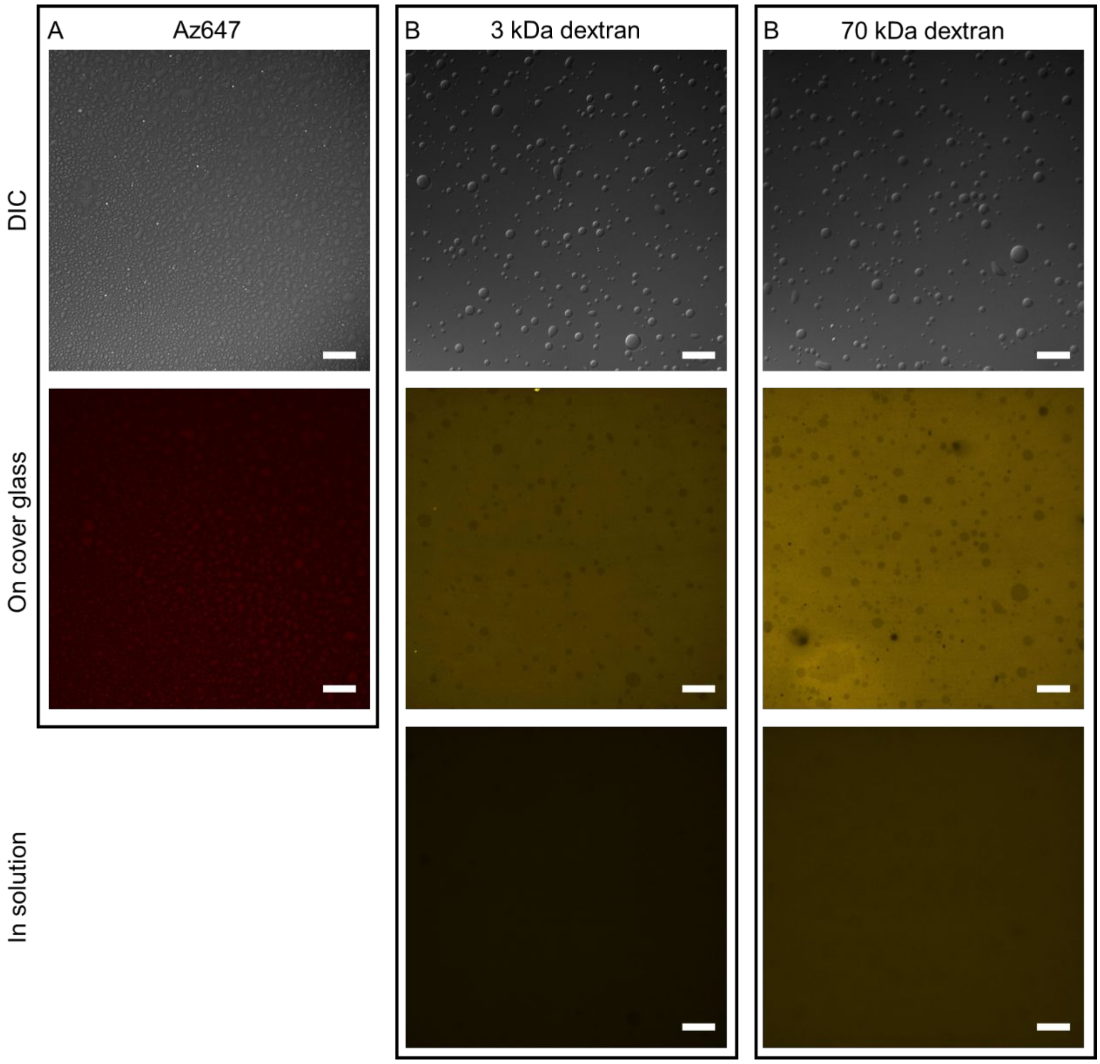

Fig. S11. Fluorescence confocal images of p-coacervate with A) Az647, B) 3 kDa dextran, C) 70 kDa dextran. The dextran showed strong affinities with the PEG coating on the cover glass. Thus, fluorescence intensities in solution were used as background to compare with intensities in coacervates. The controls were prepared in an identical manner to the samples (see Figure 5). All scale bars = 20 µm.

	Az647 Dye	3kDa Dextran	70kDa Dextran
Inside coacervate	232.5±23.3↑	376.9±33.0↑	197.6±20.1↑
In solution	162.4±15.6	348.3±32.5	185.0±18.9
On cover glass	-	463.5±37.9↑	276.0±26.8↑

Table S2. The fluorescence intensity of model molecules inside coacervates, in solution, and on the cover glass. Up arrows indicated the increased fluorescence intensity compared to the solution background.

Fluorescence intensity of		Az647 Dye	3kDa Dextran	70kDa Dextran
Inside coacervate	ELP-CLP	179.8±18.6	451.9±37.2	434.8±41.9
	Model molecule	658.7±42.4↑	379.2±37.2↑	184.1±17.5↓
Coacervate surface	ELP-CLP	453.7±22.5	784.4±41.7	1201.0±68.9
	Model molecule	324.9±18.5↑	1717.9±143.0↑	328.4±16.5↑
In solution	ELP-CLP	105.1±5.5	110.1±6.7	118.2±10.1
	Model molecule	232.0±23.4	220.5±22.0	225.4±23.0

Table S3. The fluorescence intensity of ELP-CLP and different model molecules in different fractions of coacervate and solution. Up/down arrows indicated the increased/decreased fluorescence intensity compared to the solution background.

Reference:

(1) Hwang, J.; Huang, H.; Sullivan, M. O.; Kiick, K. L. Controlled Delivery of Vancomycin from Collagen-Tethered Peptide Vehicles for the Treatment of Wound Infections. *Mol. Pharm.* **2023**, *20* (3), 1696–1708. https://doi.org/10.1021/acs.molpharmaceut.2c00898.

(2) Dunshee, L. C.; Sullivan, M. O.; Kiick, K. L. Manipulation of the Dually Thermoresponsive Behavior of Peptide-based Vesicles through Modification of Collagen-like Peptide Domains. *Bioeng. Transl. Med.* **2020**, *5* (1), e10145. https://doi.org/10.1002/btm2.10145.
